# Social and Geographical Inequalities in Suicide in Japan from 1975 through 2005: A Census-Based Longitudinal Analysis

**DOI:** 10.1371/journal.pone.0063443

**Published:** 2013-05-06

**Authors:** Etsuji Suzuki, Saori Kashima, Ichiro Kawachi, S. V. Subramanian

**Affiliations:** 1 Department of Epidemiology, Graduate School of Medicine, Dentistry and Pharmaceutical Sciences, Okayama University, Okayama, Japan; 2 Department of Public Health and Health Policy, Institute of Biomedical & Health Sciences, Hiroshima University, Hiroshima, Japan; 3 Department of Social and Behavioral Sciences, Harvard School of Public Health, Boston, Massachusetts, United States of America; Zhongshan Ophthalmic Center, China

## Abstract

**Background:**

Despite advances in our understanding of the countercyclical association between economic contraction and suicide, less is known about the levels of and changes in inequalities in suicide. The authors examined social and geographical inequalities in suicide in Japan from 1975 through 2005.

**Methods:**

Based on quinquennial vital statistics and census data, the authors analyzed the entire population aged 25–64 years. The total number of suicides was 75,840 men and 30,487 women. For each sex, the authors estimated odds ratios (ORs) and 95% credible intervals (CIs) for suicide using multilevel logistic regression models with “cells” (cross-tabulated by age and occupation) at level 1, seven different years at level 2, and 47 prefectures at level 3. Prefecture-level variance was used as an estimate of geographical inequalities in suicide.

**Results:**

Adjusting for age and time-trends, the lowest odds for suicide was observed among production process and related workers (the reference group) in both sexes. The highest OR for men was 2.52 (95% CI: 2.43, 2.61) among service workers, whereas the highest OR for women was 9.24 (95% CI: 7.03, 12.13) among security workers. The degree of occupational inequalities increased among men with a striking change in the pattern. Among women, we observed a steady decline in suicide risk across all occupations, except for administrative and managerial workers and transport and communication workers. After adjusting for individual age, occupation, and time-trends, prefecture-specific ORs ranged from 0.76 (Nara Prefecture) to 1.36 (Akita Prefecture) for men and from 0.79 (Kanagawa Prefecture) to 1.22 (Akita Prefecture) for women. Geographical inequalities have increased primarily among men since 1995.

**Conclusions:**

The present findings demonstrate a striking temporal change in the pattern of social inequalities in suicide among men. Further, geographical inequalities in suicide have considerably increased across 47 prefectures, primarily among men, since 1995.

## Introduction

Every year, almost one million people worldwide die by suicide (approximately 16 per 100,000), which amounts to one death every 40 seconds [1]. Of these suicides, more than 30,000 happen in Japan (approximately 24 per 100,000), making suicide prevention a major public health challenge for the nation [2], [3]. Indeed, the number of suicides in Japan has exceeded 30,000 every year since 1998, when a sharp increase was observed from the previous year (approximately 24,300 suicides, or 16–18 per 100,000) [4]. It has been suggested that economic decline is associated with increases in the incidence of self-destructive behavior, including suicide [5], [6]. Similarly, a countercyclical association between economic contraction and suicide has been demonstrated in Japan [7], [8]. This implies that the observed sharp increase in the suicide rate might be associated with the increased economic and social insecurity resulting from a stagnant economy since the collapse of the asset bubble in the early 1990s, and especially from the Asian financial crisis of the late 1990s [9].

Despite advances in our understanding of these countercyclical effects, less is known about the levels of and changes in inequalities in suicide during economic stagnation. Two previous studies from Japan have examined the trends in occupational inequalities in suicide among men by calculating age-standardized suicide rates [10], [11]. By examining the trends from 1965 to 1995, Kagamimori et al [10] showed that agriculture, forestry and fishery workers had a markedly high suicide rate throughout the study period, and that the pattern of inequalities across occupations was relatively stable. Wada et al [11] examined the trends from 1980 to 2005, and concluded that suicide mortality increased in 2000 across all occupations, with the largest increase among administrative and managerial workers, and specialist and technical workers. These studies strongly suggest the importance of socioeconomic disparities in suicide among Japanese men of working age. However, they did not account for geographical inequalities in suicide [12], which could pose a problem given the potential interaction between individual- and contextual-level factors on suicide risk [10], [13]. In addition, like many other studies, they did not address suicide risk among women. Previous studies have suggested a range of factors associated with suicide that relate to distal as well as more proximate causes along a hypothetical etiological pathway to suicide [13]–[18]. Given this complex, multidimensional etiology, there is an interest in investigating inequalities in suicide in terms of individual socioeconomic position as well as varying social contexts.

Accordingly, in the present study, we sought to examine social and geographical inequalities in suicide mortality in Japan from 1975 through 2005. By employing a novel multilevel approach, we explored trends by simultaneously adjusting for micro- and macro-level bias. We conducted separate analyses for men and women because the relation between economic activity and suicide may be moderated by sex [9], [13]. In line with a previous study that suggested that health disparities in Japan have widened in both sexes socially as well as geographically during the last three decades [19], we hypothesized that the social and geographical inequalities in suicide increased during the economic downturns occurring since the 1990s.

## Methods

### Data

**Figure 1:**
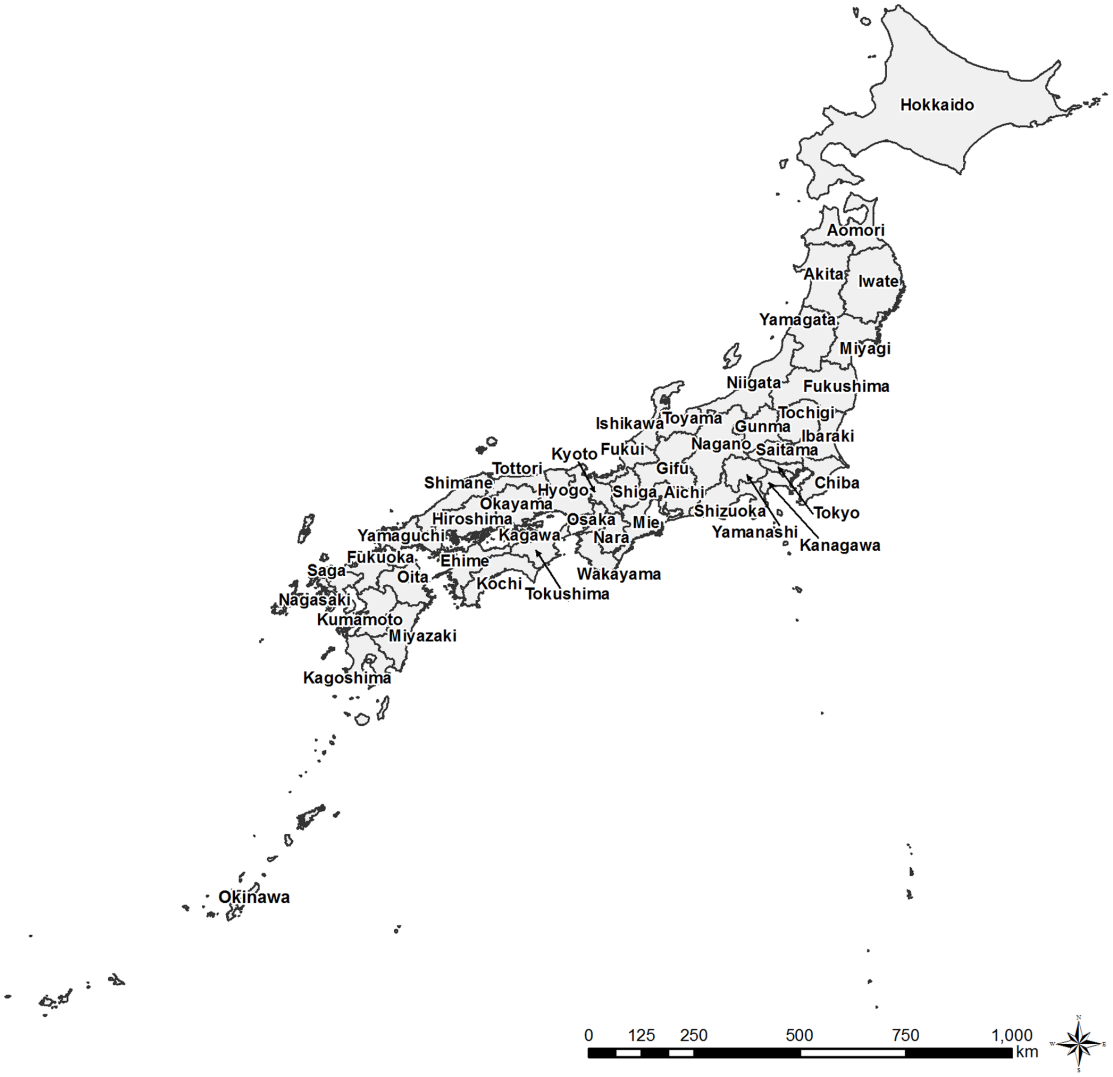
A blank map of Japan. We show the locations of 47 prefectures in Japan. Previously published in doi:10.1371/journal.pone.0039876.g001.

Suicide mortality data were obtained from the *Report of Vital Statistics: Occupational and Industrial Aspects* [20], which is compiled by the Ministry of Health, Labour and Welfare every five years, coinciding with the Population Census. Cause-specific mortality data have been available since 1975, and the latest year for which data are available is 2005. In the death notifications, respondents are asked to fill in the decedent’s occupation at the time of death [21], and one of the following persons is obliged to submit the notification: (1) relatives who lived with the decedent, (2) other housemates, (3) landlord, estate owner, land/house agent, or (4) relatives who do not live with the decedent. In the Vital Statistics, suicide was coded according to the International Classification of Disease (ICD) as follows: ICD-8 codes: E950–E959 in 1975; ICD-9 codes: E950–E959 in 1980 to 1990; and ICD-10 codes: X60–X84 in 1995 to 2005. Occupation at the time of death is recorded for each decedent following the Japan Standard Occupational Classification [22]. During the study period, the occupational classification scheme underwent four revisions (Table S1). In this study, we used the fourth revision of the Occupational Classification, which includes the following 11 groups: (1) specialist and technical workers, (2) administrative and managerial workers, (3) clerical workers, (4) sales workers, (5) service workers, (6) security workers, (7) agriculture, forestry and fishery workers, (8) transport and communication workers, (9) production process and related workers, (10) workers not classifiable by occupation, and (11) non-employed (a full description of each occupational group is available on-line [22]). Note that the group “non-employed” includes the unemployed as well as the non-labor force (e.g., home-makers, students, and the retired). Although the Census distinguishes the unemployed from home-makers, the vital records combine these categories as “non-employed.” We restricted the analysis to those who are aged 25–64 years to exclude students and the retired. The total number of suicides was 75,840 men and 30,487 women (Table 1 and Figure 1).

**Table 1:** Description of data in 47 prefectures, Japan, 1975–2005.

		Men			Women		
		No. of suicide deaths	Total population	Age-adjusted suicide rateper 100,000^a^	No. of suicide deaths	Total population	Age-adjusted suicide rate per 100,000^a^
Overall		75,840	226,497,092	33	30,487	233,031,209	13
Prefectures							
1	Hokkaido	3,901	10,234,788	38	1,452	11,093,056	13
2	Aomori	1,244	2,638,753	46	412	2,888,996	14
3	Iwate	1,175	2,537,930	45	436	2,707,295	15
4	Miyagi	1,371	4,022,987	34	512	4,162,936	12
5	Akita	1,172	2,215,679	51	418	2,405,164	17
6	Yamagata	873	2,260,858	38	313	2,348,683	13
7	Fukushima	1,345	3,755,022	35	509	3,845,709	13
8	Ibaraki	1,709	5,264,969	32	680	5,131,318	13
9	Tochigi	1,215	3,604,139	33	465	3,544,617	13
10	Gunma	1,207	3,640,335	33	550	3,645,974	15
11	Saitama	3,308	12,162,569	27	1,484	11,819,572	13
12	Chiba	3,005	10,431,257	29	1,172	10,227,688	11
13	Tokyo	6,786	22,818,497	30	3,121	22,788,213	14
14	Kanagawa	4,214	15,519,811	27	1,658	14,830,329	11
15	Niigata	1,878	4,518,024	40	676	4,626,573	14
16	Toyama	758	2,047,165	36	314	2,150,558	14
17	Ishikawa	711	2,064,408	34	262	2,184,016	12
18	Fukui	462	1,467,845	31	170	1,523,778	11
19	Yamanashi	568	1,542,329	36	213	1,560,413	13
20	Nagano	1,247	3,914,290	31	539	4,019,488	13
21	Gifu	1,145	3,720,738	30	501	3,882,407	13
22	Shizuoka	2,123	6,893,265	30	729	6,894,396	10
23	Aichi	3,489	12,745,264	27	1,489	12,493,850	12
24	Mie	944	3,254,756	28	381	3,387,226	11
25	Shiga	634	2,218,097	28	257	2,236,942	11
26	Kyoto	1,475	4,573,232	32	663	4,877,635	13
27	Osaka	5,423	16,322,242	33	2,261	16,877,110	13
28	Hyogo	3,198	9,811,455	32	1,416	10,342,041	13
29	Nara	607	2,396,422	25	310	2,571,786	12
30	Wakayama	753	1,916,177	38	326	2,079,813	15
31	Tottori	416	1,077,965	38	138	1,141,810	12
32	Shimane	621	1,363,692	44	206	1,440,197	14
33	Okayama	998	3,417,362	29	430	3,594,168	12
34	Hiroshima	1,695	5,157,004	32	715	5,369,568	13
35	Yamaguchi	1,042	2,765,322	37	453	3,026,245	14
36	Tokushima	471	1,475,947	32	212	1,575,308	13
37	Kagawa	613	1,833,497	33	258	1,934,943	13
38	Ehime	1,003	2,635,749	38	410	2,903,131	14
39	Kochi	664	1,440,043	45	231	1,575,974	14
40	Fukuoka	3,394	8,378,135	40	1,205	9,250,926	13
41	Saga	598	1,482,795	40	206	1,629,379	12
42	Nagasaki	1,108	2,655,130	41	387	2,950,956	13
43	Kumamoto	1,158	3,113,028	36	473	3,469,743	13
44	Oita	786	2,126,717	36	321	2,379,924	13
45	Miyazaki	995	2,005,522	48	340	2,219,474	15
46	Kagoshima	1,376	3,002,497	45	557	3,338,368	16
47	Okinawa	962	2,053,384	47	256	2,083,513	12

a Age-adjusted suicide rates were calculated by the direct method, using the model population of 1985 in Japan as a standard. The model population of 1985 is based on the Japanese population under census of 1985 and it is created on the basis of 1,000 persons as 1 unit, after adjusting radical increase or decrease such as baby boom.

Denominator data for the calculation of suicide rates were obtained from the Population Census which has been conducted by the Ministry of Internal Affairs and Communications every five years since 1920 [23]. In the Census questionnaire, occupation is assessed by the following question [23]: “Description of work – Describe in detail the duties you are assigned to perform.” One questionnaire is delivered to every household, and one person in each household completes it on behalf of the household members. We used “production process and related workers” as the referent category because they were the largest occupational category in the majority of the time periods (Table S2). Note that 40–50% of women were classified as the non-employed.

### Analysis

The data had a three-level structure of 28,876 cells for men and 28,843 cells for women at level 1, nested within seven years at level 2, which were nested within 47 prefectures at level 3. The seven years comprised 1975, 1980, 1985, 1990, 1995, 2000, and 2005. Each year had a maximum of 88 cells (8 age groups × 11 occupational groups) (Table S3). Note that the number of suicides in each cell was recorded during one fiscal year.

For descriptive purposes, we first calculated age-adjusted suicide rates in each occupation by using the direct method. We then employed multilevel statistical procedures because of their ability to model complex variance structures at multiple levels [24]. The unit of analysis was “cells,” and our models were structurally identical to models with individuals at level 1 [25]. The response variable, proportion of suicide deaths in each cell, was modeled with allowances made for the varying denominator in each cell. To fit multilevel binomial logit link models, we used Bayesian estimation procedures as implemented via Markov chain Monte Carlo methods by using MLwiN 2.25 [26], [27]. We used default diffuse priors for all the parameters. We obtained maximum-likelihood estimates for starting values of the distribution, then 500 simulations as discarded burn-in, then 50,000 further simulations to get the distribution of interest. Results are presented as odds ratios (ORs) and 95% credible intervals (CIs). A *P* value of less than 0.05 (two-sided) was considered statistically significant.

First, we conducted a three-level analysis as an overall model, with cells at level 1, years at level 2, and prefectures at level 3, which allows us to examine the overall patterns of occupational and geographical inequalities in suicide by adjusting for age and time-trends. The prefecture-level variance was used as an estimate of geographical inequalities in suicide. Prefectures were ranked by ORs, with the reference being the grand mean of all prefectures (value = 1), and uncertainty was estimated by 95% CIs. We also conducted a stratified analysis by age groups (25–44 years vs. 45–64 years). Subsequently, to examine the temporal patterns of occupational and geographical inequalities in suicide, we also conducted a two-level analysis, with cells at level 1 and prefectures at level 2 for each year separately.

Then, to further explore the temporal changes in occupational inequality, we ran a three-level model including a fixed cross-level interaction effect between the 11 occupations (at level 1) and year (at level 2). In this analysis, we modeled the year as a continuous variable, and we calculated mean predicted probabilities of suicide among those aged 25 to 29 (the reference category for age).

To present the geographical inequalities in suicide, we created maps showing prefecture-level residuals by using ArcGIS (ESRI Japan Inc., version 10.0).

## Results

### Social Inequalities in Suicide

Table 2 shows time-trends in occupation-specific as well as overall age-adjusted suicide rates. We also show the trends across eight regions (Table S4), which implies that occupational patterns are relatively generalizable across the whole country. High suicide rates among workers not classifiable by occupation may be an artifact due to difficulties with classifying occupations at the time of death. Table 3 shows the results of our multilevel analyses of social inequalities in suicide in terms of occupation from the overall model as well as year-specific models. Excluding workers not classifiable by occupation and the non-employed, there were substantial suicide disparities by occupation in both sexes, especially among women. Adjusting for age and time-trends in the overall model, the lowest odds for suicide were observed among production process and related workers (the reference category) in both sexes. The highest OR in men was 2.52 (95% CI: 2.43, 2.61) among service workers, whereas the highest OR in women was 9.24 (95% CI: 7.03, 12.13) among security workers. In the age-stratified analysis, the overall patterns of occupational inequalities were comparable across the age groups, except for the following three occupations among men; administrative and managerial workers (OR in 25–44 years: 0.88 (95% CI: 0.81, 0.96) vs. OR in 45–64 years: 1.09 (95% CI: 1.03, 1.14)), clerical workers (OR in 25–44 years: 1.18 (95% CI: 1.13, 1.24) vs. OR in 45–64 years: 0.98 (95% CI: 0.94, 1.03)), and sales workers (OR in 25–44 years: 0.93 (95% CI: 0.89, 0.97) vs. OR in 45–64 years: 1.34 (95% CI: 1.28, 1.39)).

**Table 2:** Age-adjusted suicide rates per 100,000 in each occupation, Japan, 1975–2005^a^.

	1975	1980	1985	1990	1995	2000	2005
*Men*							
Overall	27	28	36	25	28	44	40
Specialist and technical workers	12	19	20	14	16	38	34
Administrative and managerial workers	12	12	20	13	14	37	44
Clerical workers	18	18	22	17	17	20	17
Sales workers	22	24	30	14	14	21	18
Service workers	21	32	38	34	40	64	64
Security workers	12	14	23	18	15	27	35
Agriculture, forestry and fishery workers	34	40	59	47	48	60	59
Transport and communication workers	20	23	26	20	22	34	36
Production process and related workers	19	18	22	14	15	17	17
Workers not classifiable by occupation	1,417	1,875	1,090	420	658	669	115
Non-employed^b^	177	172	184	167	155	195	201
*Women*							
Overall	15	14	14	12	11	13	11
Specialist and technical workers	12	10	7	8	5	7	6
Administrative and managerial workers	30	10	20	32	25	29	18
Clerical workers	8	6	5	5	4	3	3
Sales workers	8	9	9	7	5	6	5
Service workers	9	11	9	8	7	9	7
Security workers	41	30	164	19	50	29	24
Agriculture, forestry and fishery workers	17	16	19	16	12	12	7
Transport and communication workers	16	29	42	31	22	24	29
Production process and related workers	7	5	6	5	3	3	3
Workers not classifiable by occupation	88	534	281	216	238	208	28
Non-employed^b^	20	18	20	18	17	22	21

a Age-adjusted suicide rates were calculated by the direct method, using the model population of 1985 in Japan as a standard. The model population of 1985 is based on the Japanese population under census of 1985 and it is created on the basis of 1,000 persons as 1 unit, after adjusting radical increase or decrease such as baby boom.

b Non-employed includes the unemployed as well as the non-labor force.

**Table 3:** Odds ratios for suicide mortality in each occupation, Japan, 1975–2005^a^

	Overall		1975		1980		1985		1990		1995		2000			2005		
	OR	95% CI	OR	95% CI	OR	95% CI	OR	95% CI	OR	95% CI	OR	95% CI		OR	95% CI		OR	95% CI
*Men*																		
Specialist and technical workers	1.27	1.23, 1.31	0.62	0.54, 0.71	0.99	0.89, 1.10	0.90	0.82, 0.99	0.93	0.84, 1.04	1.01	0.92, 1.12		2.19	2.04, 2.34		1.98	1.84, 2.13
Administrative and managerial workers	1.05	1.00, 1.09	0.62	0.54, 0.71	0.67	0.59, 0.76	0.93	0.83, 1.03	0.83	0.72, 0.95	0.93	0.82, 1.04		2.10	1.91, 2.30		2.04	1.82, 2.27
Clerical workers	1.08	1.04, 1.11	0.94	0.86, 1.02	1.02	0.93, 1.11	1.06	0.98, 1.15	1.20	1.10, 1.32	1.14	1.04, 1.25		1.22	1.12, 1.32		1.01	0.93, 1.10
Sales workers	1.11	1.08, 1.14	1.04	0.95, 1.13	1.18	1.09, 1.27	1.30	1.21, 1.40	0.91	0.83, 1.00	0.94	0.86, 1.03		1.28	1.19, 1.38		1.09	1.01, 1.18
Service workers	2.52	2.43, 2.61	1.07	0.92, 1.24	1.69	1.50, 1.91	1.75	1.57, 1.94	2.39	2.14, 2.67	2.74	2.49, 3.03		3.97	3.66, 4.29		3.78	3.48, 4.10
Security workers	1.21	1.14, 1.29	0.64	0.49, 0.82	0.76	0.61, 0.95	1.07	0.90, 1.27	1.26	1.04, 1.52	1.01	0.84, 1.23		1.55	1.34, 1.79		2.00	1.77, 2.27
Agriculture, forestry and fishery workers	2.48	2.40, 2.56	1.83	1.69, 1.99	2.08	1.92, 2.26	2.48	2.31, 2.67	3.28	3.00, 3.59	2.88	2.62, 3.18		3.26	2.96, 3.59		3.20	2.90, 3.54
Transport and communication workers	1.46	1.41, 1.52	1.02	0.92, 1.14	1.17	1.06, 1.30	1.21	1.11, 1.33	1.33	1.19, 1.48	1.42	1.28, 1.58		2.02	1.85, 2.20		2.02	1.84, 2.21
Production process and related workers	1.00	Reference	1.00	Reference	1.00	Reference	1.00	Reference	1.00	Reference	1.00	Reference		1.00	Reference		1.00	Reference
Workers not classifiable by occupation	26.95	26.06, 27.88	75.73	65.19, 87.97	109.24	97.85, 121.95	55.24	50.21, 60.77	30.75	27.68, 34.16	48.91	45.05, 53.10		43.96	41.18, 46.92		7.36	6.69, 8.11
Non-employed	9.09	8.89, 9.29	8.78	8.24, 9.37	8.73	8.20, 9.29	7.59	7.18, 8.03	10.18	9.55, 10.85	9.18	8.64, 9.76		10.55	10.01, 11.13		11.03	10.44, 11.65
*Women*																		
Specialist and technical workers	1.64	1.52, 1.78	1.72	1.39, 2.14	1.87	1.50, 2.31	1.25	1.01, 1.55	1.66	1.36, 2.03	1.91	1.49, 2.43		2.18	1.76, 2.70		2.22	1.75, 2.80
Administrative and managerial workers	4.85	4.23, 5.55	4.08	2.79, 5.97	2.04	1.24, 3.36	2.63	1.78, 3.88	4.86	3.54, 6.68	7.50	5.33, 10.55		9.18	6.60, 12.77		8.61	5.73, 12.93
Clerical workers	1.09	1.01, 1.17	1.34	1.12, 1.61	1.29	1.06, 1.57	0.92	0.77, 1.10	1.02	0.85, 1.22	1.41	1.13, 1.76		1.19	0.97, 1.47		1.14	0.90, 1.44
Sales workers	1.61	1.50, 1.74	1.24	1.02, 1.50	1.88	1.56, 2.26	1.47	1.23, 1.75	1.51	1.24, 1.83	1.78	1.40, 2.26		1.96	1.56, 2.45		1.86	1.44, 2.41
Service workers	1.91	1.78, 2.06	1.40	1.16, 1.69	2.16	1.79, 2.61	1.51	1.26, 1.82	1.65	1.36, 2.00	2.46	1.97, 3.08		2.61	2.12, 3.21		2.67	2.12, 3.36
Security workers	9.24	7.03, 12.13	4.84	1.41, 16.63	5.50	1.58, 19.11	23.90	14.33, 39.87	3.59	1.04, 12.39	17.84	9.51, 33.49		8.44	4.36, 16.34		8.53	4.40, 16.52
Agriculture, forestry and fishery workers	3.40	3.17, 3.64	2.52	2.17, 2.93	3.19	2.71, 3.76	2.91	2.51, 3.39	3.31	2.77, 3.95	4.52	3.58, 5.72		4.45	3.50, 5.64		3.69	2.71, 5.04
Transport and communication workers	5.86	4.99, 6.88	2.73	1.69, 4.39	4.37	2.82, 6.79	6.32	4.43, 9.01	5.52	3.54, 8.60	7.75	4.75, 12.62		7.20	4.54, 11.43		11.37	7.39, 17.51
Production process and related workers	1.00	Reference	1.00	Reference	1.00	Reference	1.00	Reference	1.00	Reference	1.00	Reference		1.00	Reference		1.00	Reference
Workers not classifiable by occupation	38.96	36.16, 41.98	13.82	9.52, 20.07	112.35	90.10, 140.09	50.28	41.74, 60.56	47.06	38.69, 57.22	96.49	78.44, 118.69		66.90	55.41, 80.78		11.08	8.41, 14.59
Non-employed	4.62	4.38, 4.87	2.94	2.57, 3.35	3.70	3.22, 4.25	3.63	3.22, 4.08	4.03	3.53, 4.61	6.87	5.77, 8.18		7.15	6.05, 8.45		8.16	6.74, 9.89

CI; credible interval, OR; odds ratio

a We adjusted for age (five year categories) and year in the overall model. We adjusted for only age (five year categories) in year-specific models.

**Figure 2:**
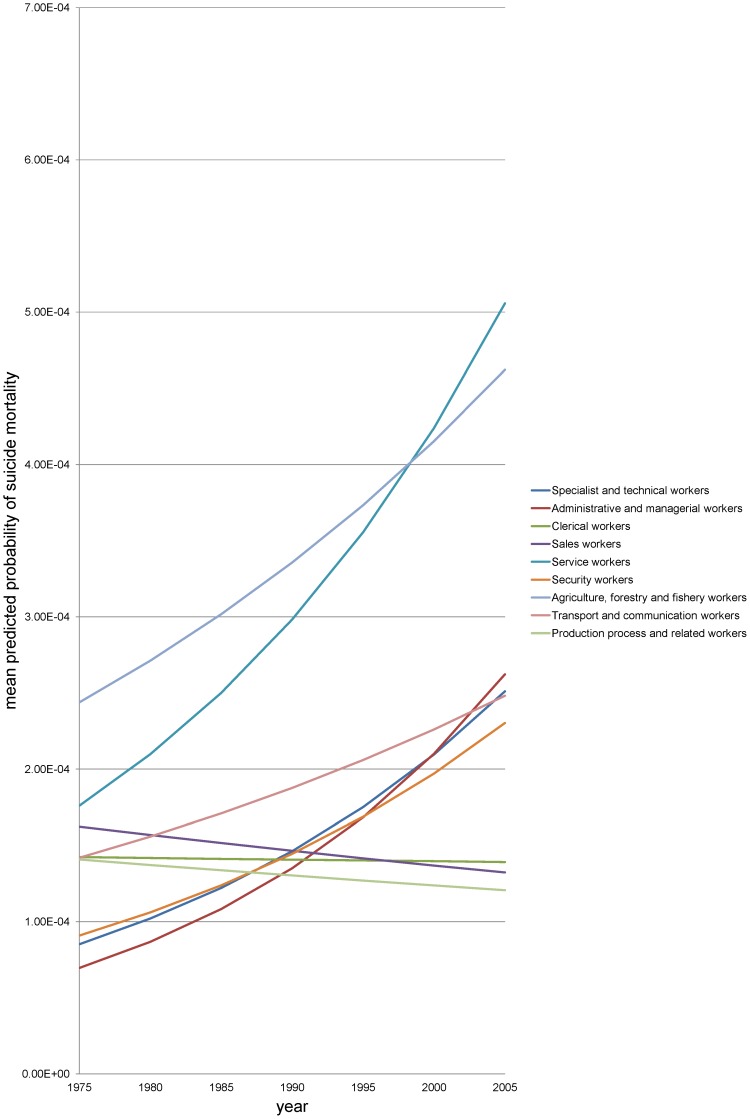
Predicted suicide mortality by occupation in men, Japan, 1975–2005. We show mean predicted probabilities of suicide mortality for nine occupational groups among those aged 25**–**29 years (the reference category for age). We excluded workers not classifiable by occupation and the non-employed from the figure to enhance readability although they were included in the analysis.

**Figure 3:**
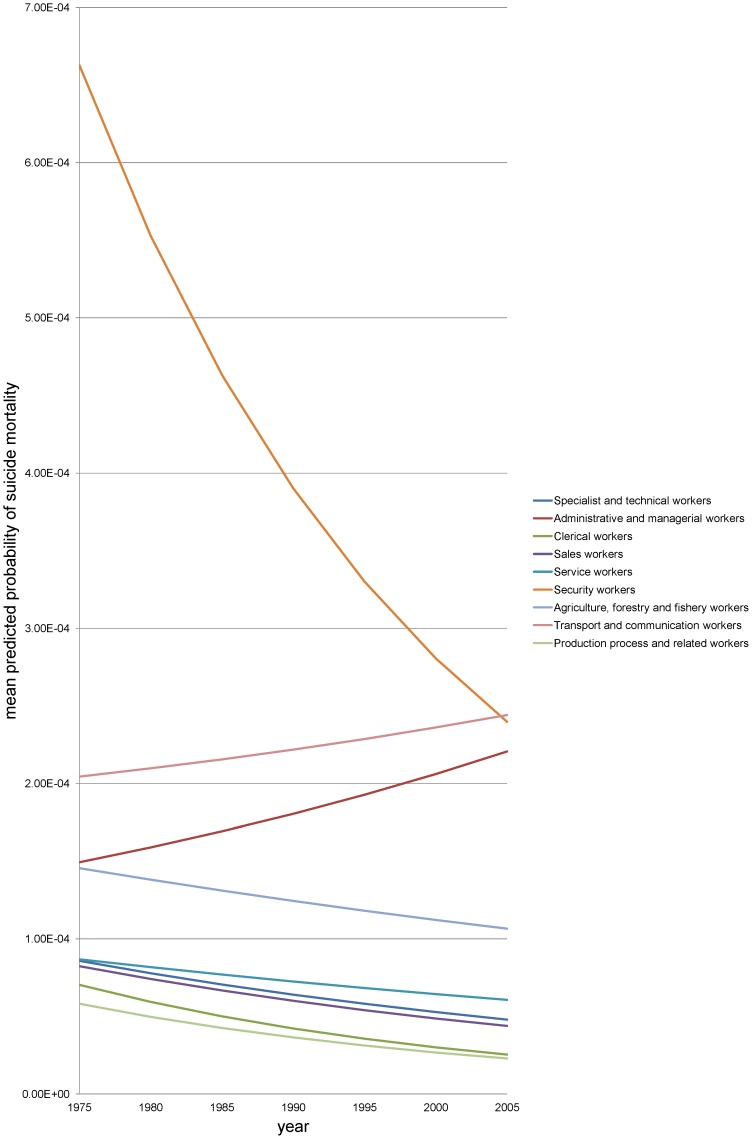
Predicted suicide mortality by occupation in women, Japan, 1975–2005. We show mean predicted probabilities of suicide mortality for nine occupational groups among those aged 25**–**29 years (the reference category for age). We excluded workers not classifiable by occupation and the non-employed from the figure to enhance readability although they were included in the analysis.

The results from year-specific models show remarkable changes in occupational inequalities in suicide risk throughout the study period among men. Figures 2 and 3 display the temporal pattern of these occupational inequalities. Among men (Figure 2), we observed a steep increase in suicide risk in some occupations, including specialist and technical workers, administrative and managerial workers, and security workers. These three occupations, having the lowest suicide risks in 1975, experienced a considerable increase in suicide risk since then. We also note that service workers and agriculture, forestry and fishery workers consistently had the highest suicide risk throughout the study period, going from bad to worse. The suicide risk among transport and communication workers moderately increased. By contrast, the suicide risk among clerical workers, sales workers, and production process and related workers remained unchanged, and persons in these occupations ended up having the lowest suicide risk in 2005.

By contrast, the pattern of social inequalities in suicide was more stable for women (Figure 3). Most occupations experienced comparable trajectories with a steady decline in suicide with the exception of administrative and managerial workers – they experienced a small increase in suicide risk. The suicide risk among transport and communication workers also slightly increased, and they ended up having the highest risk in 2005.

### Geographical Inequalities in Suicide

**Figure 4:**
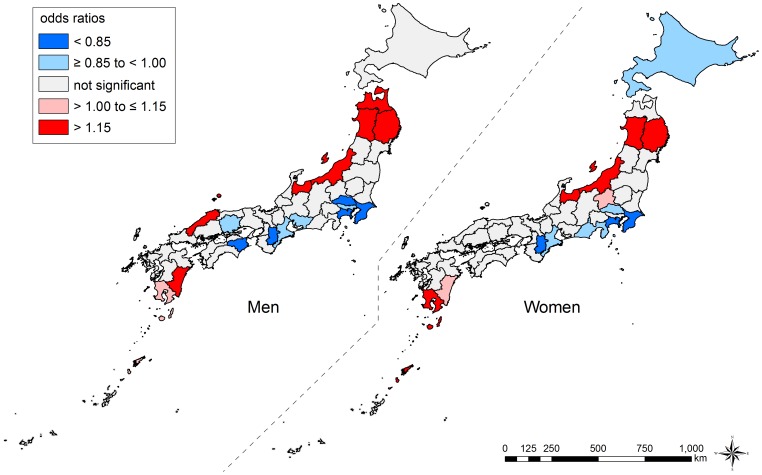
Geographical inequalities in suicide among those aged 25–64 years, Japan, 1975–2005. We show the overall geographical inequalities in suicide mortality across 47 prefectures, conditional on individual age, occupation, and time-trends. Prefecture-level residuals are described by odds ratios, with the reference being the grand mean of all prefectures. Prefectures with lower odds for suicide are blue, and those with higher odds are red. The prefectures with non-significant residuals are gray.

Conditional on individual age, occupation, and time-trends, the overall geographical inequalities in suicide were more salient in men than in women (Table 4). Prefecture-specific ORs ranged from 0.76 (Nara Prefecture) to 1.36 (Akita Prefecture) for men and from 0.79 (Kanagawa Prefecture) to 1.22 (Akita Prefecture) for women (Tables S5 and S6). Figure 4 displays the geographical inequalities in suicide. We observed similar patterns in both sexes. In the age-stratified analysis, we found no substantial differences across the age groups (data not shown).

**Table 4:** Adjusted prefecture-level variance for suicide mortality, Japan, 1975–2005^a^.

	Men			Women		
	Variance (on logit scale)			Variance (on logit scale)		
	Estimate	95% CI	Range of OR b	Estimate	95% CI	Range of OR b
Overall	0.018	0.009, 0.028	0.76 to 1.36	0.012	0.006, 0.019	0.79 to 1.22
1975	0.010	0.002, 0.017	0.89 to 1.16	0.011	0.002, 0.021	0.86 to 1.20
1980	0.016	0.006, 0.027	0.75 to 1.30	0.012	0.003, 0.022	0.77 to 1.14
1985	0.019	0.008, 0.029	0.82 to 1.33	0.028	0.010, 0.046	0.76 to 1.28
1990	0.019	0.007, 0.030	0.83 to 1.32	0.016	0.003, 0.029	0.84 to 1.23
1995	0.045	0.023, 0.068	0.63 to 1.64	0.031	0.011, 0.052	0.76 to 1.29
2000	0.033	0.017, 0.050	0.71 to 1.54	0.019	0.004, 0.033	0.80 to 1.26
2005	0.051	0.028, 0.075	0.67 to 1.64	0.016	0.003, 0.030	0.80 to 1.21

CI; credible interval, OR; odds ratio.

a We adjusted for age (five year categories) and occupation. We further adjusted for time-trends in the overall model.

b The range of adjusted odds ratios for suicide mortality in each prefecture is shown. The reference is the grand mean of all prefectures.

**Figure 5:**
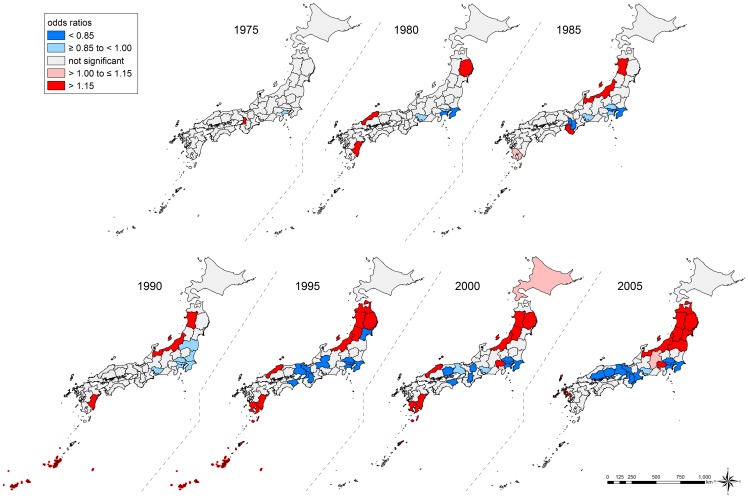
Geographical and temporal variation in suicide among men aged 25–64 years, Japan, 1975–2005. We show year-specific geographical inequalities in suicide mortality across 47 prefectures, conditional on individual age and occupation. Prefecture-level residuals are described by odds ratios with the reference being the grand mean of all prefectures. Prefectures with lower odds for suicide are blue, and those with higher odds are red. The prefectures with non-significant residuals are gray.

**Figure 6:**
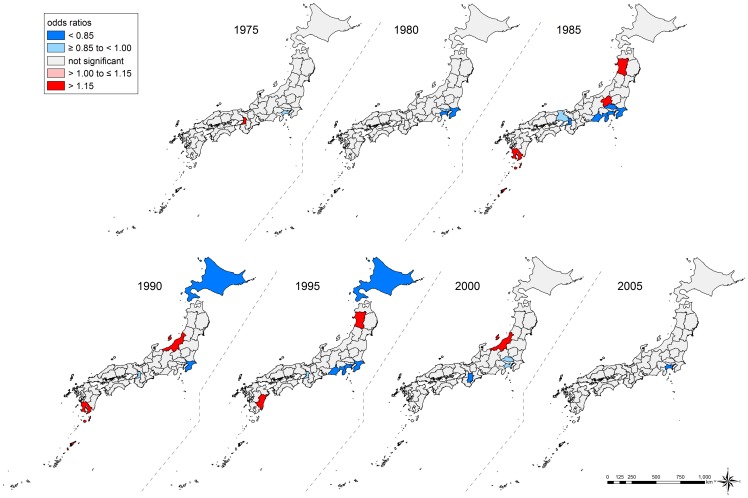
Geographical and temporal variation in suicide among women aged 25–64 years, Japan, 1975–2005. We show year-specific geographical inequality in suicide mortality across 47 prefectures, conditional on individual age and occupation. Prefecture-level residuals are described by odds ratios with the reference being the grand mean of all prefectures. Prefectures with lower odds for suicide are blue, and those with higher odds are red. The prefectures with non-significant residuals are gray.

We observed a remarkable increase in geographical inequalities in suicide among men throughout the study period (Table 4). In men, although the prefecture-level variance was lower than that for women in 1975 (0.010 on logit scale), it increased drastically up to 0.045 in 1995, followed by 0.051 in 2005, which is the highest ever throughout the period. By contrast, the increasing trend in geographical inequalities was less clear among women, and we observed rises and falls of the prefecture-level variance, including two spikes in 1985 and 1995. The adjusted ORs for suicide in each prefecture across years are shown in Tables S5 and S6. In 1975, the ORs ranged from 0.89 (Kanagawa Prefecture) to 1.16 (Osaka Prefecture) for men and from 0.86 (Kanagawa Prefecture) to 1.20 (Osaka Prefecture) for women. In 2005, the ranges were widened, with ORs from 0.67 (Nara Prefecture) to 1.64 (Akita Prefecture) for men and from 0.80 (Kanagawa Prefecture) to 1.21 (Iwate Prefecture) for women. The temporal patterns in geographical inequalities are also shown using maps, and suggest an increase since 1995 among men (Figures 5 and 6, Video S1).

## Discussion

In this study, we examined the trends in social and geographical inequalities in suicide mortality in Japan over the past three decades. The degree of occupational inequalities increased among men, and we observed a striking change in the pattern – the risk of suicide markedly increased among specialist and technical workers, administrative and managerial workers, and security workers, even though they all had the lowest suicide risk in 1975. Among men, we also note steep upward trends in suicide risk among service workers and agriculture, forestry and fishery workers. By contrast, among women, we observed a steady decline in suicide risk across all occupations, except for administrative and managerial workers and transport and communication workers. Meanwhile, across prefectures, suicide risk varied by as much as 70% and 50% among men and women, respectively, even adjusting for individual age, occupation, and time-trends. Furthermore, the geographical inequalities in suicide have increased, primarily among men, since 1995.

Previous studies have suggested that some occupational groups are at an increased risk of suicide, such as medical doctors, nurses, dentists, pharmacists, veterinary surgeons, police officers, and farmers [15], [16], [28]–[30]. These high-risk occupational groups have easy access to means for suicide [16], and they may be also reluctant to seek help because of perceived stigma and other factors [30]. However, these findings are rather inconsistent [31], [32], and the link between occupation and suicide is not well understood. As noted above, our findings demonstrate a remarkable, hitherto unrecognized change in the pattern of occupational inequalities in suicide among Japanese men – a sharp increase among some occupations and a slight decline among others – although the pattern among women was rather consistent. These findings can be interpreted within the context of the dramatic changes in the Japanese economy throughout the study period. Japan experienced a second economic crisis (the so-called secondary oil crisis) in 1980–1983 and economic prosperity (a bubble economy) in 1986–1990. Following the collapse of the asset bubble in the early 1990s, however, the Japanese economy has basically been stagnant, and around mid-1997, a series of financial crises struck East Asia and caused extremely negative effects such as a sharp decline in foreign exchange rates and stock prices as well as the bankruptcy of many companies and massive layoffs [33]. Under the lingering economic stagnation, persons in some occupations could have experienced stressful life events, including financial problems and family conflicts, which could lead to higher risk of common mental disorders [34], [35]. We note that Japanese men of working age used to have a strong commitment to the company, and this may have led to a severe challenge for them when becoming the target of corporate restructuring following the economic crisis [9]. A breakdown of social cohesion within companies, accompanied with extremely long working hours and the fear of losing one’s job, could lead to a higher risk of suicide even among those who could remain on the payroll [13], [36]–[39]. The experience of persistent stress may be a driving force behind the remarkable changes in the pattern of social inequalities in suicide among men. In line with this, a recent review explained that acute life stresses such as job loss, gambling, and work-related factors are important precipitants of suicide among Asian men, whereas family conflicts are a key risk factor for Asian women [13]. In addition, we should note that the increased risks in certain occupations may be explained by the demographic and personality characteristics of persons in those occupations. However, the evidence remains unclear about whether the selection process could fully explain the growing suicidal risk among some occupational groups.

Among women, the finding of a steady decline in suicide risk across almost all occupational groups may imply that Japanese women of working age are less vulnerable to the economic crisis compared with men [40], [41]. These findings may be also explained by differences between men and women according to type of work and industrial sector of employment. Men are more likely to be engaged in work in the private sector as well as in parts of the economy that are more vulnerable to economic downturn [42].

The present findings demonstrate a remarkably widening East-West inequality in suicide risk among men, especially since 1995. All six prefectures in the Tohoku region (the northeastern district of the main island) and the Niigata Prefecture (a neighboring prefecture to the Tohoku region) were classified as having the highest suicide risk, whereas some prefectures in the Kanto, Kinki, and Chugoku regions (central or western districts of the main island) were classified as having the lowest suicide risk. Although it has been reported that prefectures with high suicide rates are located in the Tohoku region [43], no studies have provided suggestive evidence of emerging common ecologic effects of place where people live [44] by adjusting for individual age and occupation in each prefecture. The present study applied the same adjustment strategy across 47 prefectures throughout the study period, and it is unlikely that the widening geographical inequalities simply reflect an omitted compositional effect (e.g., health behaviors such as alcohol consumption [45]–[48] and smoking [49], [50], social support [51], and living arrangement [52]). In other words, it is likely that contextual effects were behind the widening geographical inequalities, primarily among men.

There are some limitations of our analysis. First, there are concerns about the quality of cause-of-death coding and the completeness of the ascertainment of suicide cases [53]. However, death registration is mandatory in Japan and all death certificates are signed by medical doctors. If the death is certified as a suicide, the police must be notified, and suspected suicides need to be examined by a qualified medical pathologist. All death certificates are sent to the Ministry of Health, Labour and Welfare, and coded for National Vital Statistics. Thus, we believe that the quality and completeness of suicide mortality data in this study are reliable. Indeed, the quality of mortality data in Japan is rated as level-1 (best quality) by the Department of Measurement and Health Information of the World Health Organization [4]. Besides, considering that the numbers of deaths classified as undetermined intent were relatively low (Table S7), the present findings are less likely to be influenced by misclassified suicides.

Second, we should note that the present findings do not necessarily apply to the pattern of inequalities in suicidal behavior. It is important to realize that despite some similarities [54], suicide attempters and completers in Japan would have significant demographic, personality, and clinical differences [55]. Previous suicide attempts or deliberate self-harm are regarded as very important risk factors for completed suicide [18], and thus, from a perspective of preventive medicine, it would be beneficial to examine the trends in suicidal ideation and parasuicide within the nation.

Third, the smallest geographical unit available was the prefecture, and we could not explore geographical inequalities in finer detail. Further analysis using different area units would lead to a better understanding of the causal processes involved in the potential contextual effect(s) on suicide – if suicide mortality is strongly dependent on characteristics of the immediate physical and social environment, as well as psychological, behavioral and life attributes, then smaller area units would capture the right level of area differentiation. By contrast, the prefecture may be a useful and valid unit of analysis because it has direct administrative authority over the economic, education, and health sectors [56]. Furthermore, the prefecture has specific jurisdiction over health centers, which are the loci of preventive health care activity [56]. Further studies are warranted to examine the relative importance of smaller and larger area units when examining the risk of suicide.

Fourth, unaccounted individual variability and selective residential migration cannot be excluded as alternative explanations for our observed associations. Although we used relatively fine occupational groupings, we should recognize the possibility that the *composition* of each occupational group went through a (substantial) change throughout the study period. Indeed, neither employment status nor the predominant type of employment contract was available, and in particular, we lacked information on whether the individuals were in standard jobs or precarious jobs. In this regard, a more detailed assessment of occupational factors would be desirable [57].

Finally, occupation at the time of death was used in our numerator data, which may not necessarily reflect the individual’s life-course socioeconomic position [58], [59]. On a related issue, given the possible discrepancies between data from the two occasions (i.e., the notification of deaths and the Census), numerator/denominator bias between the two sources of information must be considered.

The present findings demonstrate a striking temporal change in the pattern of social inequalities in suicide among men. Further, even adjusting for individual age and occupation in each prefecture, the findings show considerable geographical disparities in suicide across the 47 prefectures in both sexes, with a striking increase among men. These dramatic changes in suicide inequalities were apparently enhanced through Japan’s long-stagnant economy. In June 2006, a fundamental law presenting a suicide prevention strategy was established in Japan. In addition, a recent review from Japan proposed a strategy for the early detection of suicide risk by screening for depression according to self-perceived symptoms in order to implement extensive interventions for high-risk individuals within these groups [60]. Given the limited effect of national suicide prevention programs on suicide rates among working-age groups in both sexes [61], however, suicide prevention strategies should focus not only on individual psychiatric factors but also on the potential effects of broader contextual factors [17], with a perspective on population-level disease burden [62].

## Supporting Information

Table S1 The history of the Japan Standard Occupational Classification.
(PDF)

Table S2 The number in each occupation and their percentage of the total population among those aged 25–64 years, Japan, 1975–2005.
(PDF)

Table S3 Description of data used for multilevel models analyzing suicide mortality in 47 prefectures, Japan, 1975–2005.
(PDF)

Table S4 Time trends in age-adjusted suicide rates per 100,000 in each occupation across eight regions, Japan, 1975–2005.
(PDF)

Table S5 Adjusted prefecture-level residuals for suicide mortality among men, Japan, 1975–2005.
(PDF)

Table S6 Adjusted prefecture-level residuals for suicide mortality among women, Japan, 1975–2005.
(PDF)

Table S7 The number of deaths classified as undetermined intent among those aged 25–64 years, Japan, 1975–2005.
(PDF)

Video S1 Geographical and temporal variation in suicide, Japan.
(MOV)
