# Supplementary material for: Social and Geographical Inequalities in Suicide in Japan from 1975 through 2005: A Census-Based Longitudinal Analysis
Source: PLoS One. 2013 May 6;8(5):e63443. doi: 10.1371/journal.pone.0063443 (PMC3646025; doi:10.1371/journal.pone.0063443)
Supplement: Table S1 — The history of the Japan Standard Occupational Classification. (PDF) [file pone.0063443.s001.pdf]

**Table S1.** The history of the Japan Standard Occupational Classification[a]

| 1st revision, 1970 | | | 2nd revision, 1979 | | | 3rd revision, 1986 | | | 4th revision, 1997 | | |
|---|---|---|---|---|---|---|---|---|---|---|---|
| **No.** | | **Occupation (major group)** | **No.** | | **Occupation (major group)** | **No.** | | **Occupation (major group)** | **No.** | | **Occupation (major group)** |
| (1) | [1] | Professional and technical workers | (1) | [1] | Professional and technical workers | (1) | [1] | Professional and technical workers | (1) | [1] | Specialist and technical workers |
| (2) | [2] | Managers and officials | (2) | [2] | Managers and officials | (2) | [2] | Managers and officials | (2) | [2] | Administrative and managerial workers |
| (3) | [3] | Clerical and related workers | (3) | [3] | Clerical and related workers | (3) | [3] | Clerical and related workers | (3) | [3] | Clerical workers |
| (4) | [4] | Sales workers | (4) | [4] | Sales workers | (4) | [4] | Sales workers | (4) | [4] | Sales workers |
| (5) | [7] | Farmers, Lumbermen and fishermen | (5) | [7] | Agricultural, forestry and fisheries workers | (5) | [5] | Service workers | (5) | [5] | Service workers |
| (6) | [9] | Workers in mining and quarrying occupations | (6) | [9] | Mining workers | (6) | [6] | Protective service workers | (6) | [6] | Security workers |
| (7) | [8] | Workers in transport and communications occupations | (7) | [8] | Workers in transport and communications occupations | (7) | [7] | Agricultural, forestry and fisheries workers | (7) | [7] | Agriculture, forestry and fishery workers |
| (8) | [9] | Craftsmen, production process workers and labourers | (8) | [9] | Craftsmen, production process workers and labours | (8) | [8] | Workers in transport and communications occupations | (8) | [8] | Transport and communication workers |
| (9) | [6] | Protective service workers | (9) | [6] | Protective service workers | (9) | [9] | Craftsmen, mining, production process and construction workers and laborers | (9) | [9] | Production process and related workers |
| (10) | [5] | Service workers | (10) | [5] | Service workers | (10) | [10] | Workers not classifiable by occupation | (10) | [10] | Workers not classifiable by occupation |
| (11) | [10] | Unclassifiable | (11) | [10] | Workers not classifiable by occupation | (11) | [11] | Non-employed [b] | (11) | [11] | Non-employed [b] |
| (12) | [11] | Non-employed [b] | (11) | [11] | Non-employed [b] | | | | | | |

[a] We consistently used occupation (major group) of the 4th revision. The number in square brackets is the classification used in ths present study.

[b] Non-employed includes the unemployed as well as the non-labor force in line with the *Report of Vital Statistics: Occupational and Industrial Aspects* .
