# Supplementary material for: Social and Geographical Inequalities in Suicide in Japan from 1975 through 2005: A Census-Based Longitudinal Analysis
Source: PLoS One. 2013 May 6;8(5):e63443. doi: 10.1371/journal.pone.0063443 (PMC3646025; doi:10.1371/journal.pone.0063443)
Supplement: Table S2 — The number in each occupation and their percentage of the total population among those aged 25–64 years, Japan, 1975–2005. (PDF) [file pone.0063443.s002.pdf]

Table S2. The number in each occupation and their percentage of the total population among those aged 25–64 years, Japan, 1975–2005

	1975		1980		1985		1990		1995		2000		2005	
	N	%	N	%	N	%	N	%	N	%	N	%	N	%
Men														
Specialist and technical workers	2,080,025	7.25	2,306,830	7.51	3,143,412	9.76	3,637,515	10.95	3,991,077	11.72	4,221,683	12.27	3,950,815	11.90
Administrative and managerial workers	1,972,340	6.88	2,210,783	7.19	1,868,101	5.80	1,998,511	6.01	2,066,172	6.07	1,305,093	3.79	1,031,316	3.11
Clerical workers	3,674,725	12.81	3,637,048	11.83	3,857,022	11.98	3,895,784	11.72	3,906,006	11.47	4,077,310	11.85	4,093,124	12.33
Sales workers	3,508,340	12.23	4,132,015	13.44	4,509,884	14.00	4,794,455	14.43	5,044,836	14.82	5,159,661	15.00	4,716,064	14.21
Service workers	984,940	3.43	1,027,910	3.34	1,123,385	3.49	1,202,319	3.62	1,270,668	3.73	1,381,504	4.02	1,441,522	4.34
Security workers	520,720	1.82	567,438	1.85	615,053	1.91	660,161	1.99	706,462	2.08	787,325	2.29	832,148	2.51
Agriculture, forestry and fishery workers	2,849,180	9.94	2,379,666	7.74	2,112,513	6.56	1,615,756	4.86	1,199,620	3.52	899,881	2.62	823,066	2.48
Transport and communication workers	1,972,390	6.88	2,072,133	6.74	1,997,137	6.20	1,984,890	5.97	2,020,393	5.93	1,957,847	5.69	1,794,551	5.41
Production process and related workers	9,645,620	33.63	10,682,007	34.76	10,644,436	33.05	10,985,461	33.06	10,945,330	32.15	10,762,241	31.28	10,451,026	31.48
Workers not classifiable by occupation	13,870	0.05	22,474	0.07	50,391	0.16	115,015	0.35	151,362	0.44	294,663	0.86	502,667	1.51
Non-employed [a]	1,456,032	5.08	1,696,114	5.52	2,283,403	7.09	2,339,703	7.04	2,744,327	8.06	3,559,611	10.35	3,559,611	10.72
Total	28,678,182	100.00	30,734,418	100.00	32,204,737	100.00	33,229,570	100.00	34,046,253	100.00	34,406,819	100.00	33,195,910	100.00
Women														
Specialist and technical workers	1,121,045	3.73	1,507,610	4.72	1,891,400	5.73	2,250,231	6.69	2,684,971	7.83	3,094,599	8.87	3,459,894	9.83
Administrative and managerial workers	105,985	0.35	155,251	0.49	171,782	0.52	184,219	0.55	199,894	0.58	142,983	0.41	123,283	0.35
Clerical workers	2,753,760	9.16	3,369,822	10.56	4,248,922	12.86	5,155,485	15.32	5,748,954	16.76	6,289,031	18.03	6,422,961	18.25
Sales workers	2,152,320	7.16	2,586,857	8.11	2,447,212	7.41	2,534,197	7.53	2,702,863	7.88	2,618,387	7.51	2,561,132	7.28
Service workers	1,974,925	6.57	2,106,305	6.60	2,173,931	6.58	2,263,285	6.73	2,516,848	7.34	2,825,178	8.10	3,207,147	9.11
Security workers	8,010	0.03	9,876	0.03	12,390	0.04	16,562	0.05	24,289	0.07	37,414	0.11	43,158	0.12
Agriculture, forestry and fishery workers	3,154,040	10.49	2,471,427	7.75	2,029,368	6.14	1,478,304	4.39	1,055,672	3.08	755,524	2.17	600,419	1.71
Transport and communication workers	108,500	0.36	108,205	0.34	96,205	0.29	84,717	0.25	93,936	0.27	92,226	0.26	85,394	0.24
Production process and related workers	3,691,205	12.28	4,456,927	13.97	4,911,261	14.87	5,158,278	15.33	4,862,147	14.17	4,664,292	13.37	4,228,532	12.01
Workers not classifiable by occupation	34,995	0.12	24,186	0.08	66,917	0.20	89,544	0.27	121,135	0.35	223,913	0.64	327,266	0.93
Non-employed [a]	14,949,973	49.74	15,110,843	47.36	14,978,370	45.35	14,434,745	42.90	14,296,062	41.67	14,141,088	40.54	14,141,088	40.17
Total	30,054,758	100.00	31,907,309	100.00	33,027,758	100.00	33,649,567	100.00	34,306,771	100.00	34,884,635	100.00	35,200,274	100.00

[a] Non-employed includes the unemployed as well as the non-labor force.
