# Supplementary material for: Social and Geographical Inequalities in Suicide in Japan from 1975 through 2005: A Census-Based Longitudinal Analysis
Source: PLoS One. 2013 May 6;8(5):e63443. doi: 10.1371/journal.pone.0063443 (PMC3646025; doi:10.1371/journal.pone.0063443)
Supplement: Table S3 — Description of data used for multilevel models analyzing suicide mortality in 47 prefectures, Japan, 1975–2005. (PDF) [file pone.0063443.s003.pdf]

Table S3. Description of data used for multilevel models analyzing suicide mortality in 47 prefectures, Japan, 1975–2005

Characteristics	Men					Women				
	No. of cells [a]	No. of suicide deaths	Total population	Suicide rate per 100,000 [b]	(SD)	No. of cells [a]	No. of suicide deaths	Total population	Suicide rate per 100,000 [b]	(SD)
Overall	28,876	75,840	226,497,092	209	(1,742)	28,843	30,487	233,031,209	55	(660)
Level 1: cell										
Specialist and technical workers										
25-29 y	329	530	3,888,159	17	(21)	329	195	3,572,393	6	(10)
30-34 y	329	542	4,066,756	15	(19)	329	156	2,659,697	6	(13)
35-39 y	329	591	3,762,304	17	(20)	329	142	2,417,547	6	(14)
40-44 y	329	620	3,357,225	22	(23)	329	150	2,305,628	7	(14)
45-49 y	329	769	2,902,665	29	(28)	329	122	2,033,537	6	(14)
50-54 y	329	809	2,391,228	33	(38)	329	126	1,567,904	9	(18)
55-59 y	329	755	1,877,231	39	(48)	329	99	1,004,569	11	(31)
60-64 y	329	396	1,085,789	37	(54)	329	59	448,475	16	(52)
Administrative and managerial workers										
25-29 y	329	48	218,875	29	(127)	328	6	22,719	16	(157)
30-34 y	329	81	622,793	20	(74)	329	15	50,667	46	(305)
35-39 y	329	135	1,187,754	14	(36)	329	23	92,555	19	(92)
40-44 y	329	289	1,824,130	22	(34)	329	23	141,961	10	(48)
45-49 y	329	448	2,297,120	24	(35)	329	37	188,011	17	(79)
50-54 y	329	580	2,512,706	28	(34)	329	68	213,688	20	(70)
55-59 y	329	573	2,285,689	28	(34)	329	40	207,309	20	(76)
60-64 y	329	291	1,503,249	21	(32)	329	33	166,487	21	(98)
Clerical workers										
25-29 y	329	671	3,960,737	18	(18)	329	352	6,860,889	6	(9)
30-34 y	329	648	4,173,085	18	(18)	329	208	5,135,278	4	(7)
35-39 y	329	675	4,085,799	17	(16)	329	191	4,982,773	4	(9)
40-44 y	329	751	3,933,676	21	(20)	329	193	5,121,861	4	(9)
45-49 y	329	837	3,700,663	24	(23)	329	214	4,660,897	5	(9)
50-54 y	329	792	3,302,327	24	(22)	329	171	3,677,102	5	(9)
55-59 y	329	512	2,619,470	20	(23)	329	122	2,423,883	6	(19)
60-64 y	329	170	1,365,262	13	(27)	329	36	1,126,252	4	(15)
Sales workers										
25-29 y	329	562	5,235,044	13	(15)	329	143	2,073,531	8	(16)
30-34 y	329	599	5,383,356	13	(15)	329	139	1,971,428	6	(14)
35-39 y	329	692	5,003,992	18	(18)	329	124	2,294,947	5	(12)
40-44 y	329	794	4,520,363	22	(22)	329	156	2,646,474	6	(10)
45-49 y	329	1,005	3,970,321	32	(30)	329	185	2,759,364	7	(12)
50-54 y	329	988	3,367,461	37	(32)	329	210	2,533,410	9	(15)
55-59 y	329	848	2,685,924	37	(36)	329	156	2,010,056	9	(16)
60-64 y	329	511	1,698,794	34	(38)	329	115	1,313,758	9	(20)
Service workers										
25-29 y	329	375	1,485,277	29	(35)	329	202	1,689,226	14	(24)
30-34 y	329	358	1,312,656	29	(40)	329	175	1,733,370	9	(16)
35-39 y	329	383	1,176,337	39	(50)	329	155	2,116,096	8	(14)
40-44 y	329	461	1,078,948	48	(55)	329	176	2,509,611	7	(13)
45-49 y	329	561	983,175	65	(75)	329	201	2,704,363	9	(14)
50-54 y	329	595	894,930	64	(77)	329	209	2,677,366	8	(13)
55-59 y	329	558	820,949	64	(76)	329	193	2,277,148	9	(17)
60-64 y	329	300	679,976	42	(73)	329	119	1,360,439	9	(22)
Security workers										
25-29 y	329	118	749,368	15	(37)	326	7	38,006	23	(263)
30-34 y	329	111	683,447	18	(44)	320	7	23,235	17	(158)
35-39 y	329	113	629,215	17	(38)	321	7	17,446	181	(2,806)
40-44 y	329	122	615,612	16	(32)	323	9	17,797	110	(853)
45-49 y	329	181	621,942	32	(67)	326	5	17,799	27	(283)
50-54 y	329	158	575,320	27	(55)	321	8	17,028	36	(363)
55-59 y	329	132	484,783	26	(58)	313	9	13,567	41	(344)
60-64 y	329	67	329,620	20	(74)	300	4	6,821	159	(1,674)
Agriculture, forestry and fishery workers										
25-29 y	329	293	683,466	46	(80)	329	75	442,656	13	(56)
30-34 y	329	377	805,280	52	(75)	329	82	711,465	13	(53)
35-39 y	329	425	999,264	46	(54)	329	127	1,018,905	8	(18)
40-44 y	329	627	1,307,945	53	(58)	329	206	1,367,176	13	(24)
45-49 y	329	802	1,648,817	59	(55)	329	303	1,746,779	18	(37)
50-54 y	329	919	1,878,069	54	(47)	329	390	2,028,779	18	(24)
55-59 y	329	1,011	2,108,375	50	(38)	329	396	2,149,926	19	(25)
60-64 y	329	999	2,448,466	42	(33)	329	393	2,079,068	18	(22)
Transport and communication workers										
25-29 y	329	303	1,690,514	19	(33)	329	21	124,283	19	(130)
30-34 y	329	387	1,985,796	19	(25)	329	21	104,743	17	(88)
35-39 y	329	440	2,077,538	23	(29)	329	25	104,714	17	(73)
40-44 y	329	597	2,101,562	30	(32)	329	21	109,123	13	(69)
45-49 y	329	616	2,037,400	32	(32)	329	24	99,525	23	(114)
50-54 y	329	605	1,841,475	32	(33)	329	27	72,778	28	(134)
55-59 y	329	517	1,413,495	32	(39)	328	18	40,547	27	(164)
60-64 y	329	168	651,561	23	(59)	318	5	13,470	39	(433)
Production process and related workers										
25-29 y	329	1,361	10,692,627	13	(10)	329	129	2,309,022	5	(10)
30-34 y	329	1,460	10,755,951	15	(11)	329	136	3,067,564	4	(8)
35-39 y	329	1,560	10,396,964	17	(11)	329	161	4,295,460	4	(7)
40-44 y	329	1,827	10,265,445	20	(14)	329	197	5,281,703	4	(7)
45-49 y	329	2,144	10,010,317	24	(14)	329	230	5,528,330	4	(6)
50-54 y	329	2,156	9,332,509	26	(15)	329	272	5,112,741	6	(8)
55-59 y	329	1,692	7,897,054	24	(17)	329	191	4,033,500	5	(8)
60-64 y	329	829	4,765,254	18	(17)	329	108	2,344,322	5	(10)
Workers not classifiable by occupation										
25-29 y	323	516	205,041	1,673	(5,428)	328	182	149,785	447	(1,870)
30-34 y	323	499	179,894	1,282	(4,405)	329	176	121,600	260	(907)
35-39 y	314	504	149,833	1,973	(5,568)	329	131	110,990	255	(913)
40-44 y	320	577	135,486	2,528	(8,216)	328	139	115,968	282	(858)
45-49 y	322	779	128,236	2,173	(5,481)	327	181	116,471	348	(1,413)
50-54 y	315	905	131,629	2,434	(6,610)	327	231	111,303	413	(1,336)
55-59 y	318	759	125,616	1,881	(4,016)	325	186	96,021	504	(1,882)
60-64 y	321	489	95,910	1,428	(3,246)	325	143	65,955	848	(3,471)
Non-employed										
25-29 y	329	2,584	2,226,616	136	(79)	329	2,063	13,781,587	16	(10)
30-34 y	329	2,450	1,473,332	177	(94)	329	2,254	15,746,209	16	(9)
35-39 y	329	2,427	1,250,641	196	(116)	329	2,051	13,237,613	17	(10)
40-44 y	329	2,716	1,222,792	222	(106)	329	2,138	10,812,226	23	(14)
45-49 y	329	3,192	1,382,608	224	(108)	329	2,379	10,360,614	27	(15)
50-54 y	329	3,699	1,610,040	206	(104)	329	2,855	11,319,968	28	(14)
55-59 y	329	4,273	2,358,010	164	(98)	329	3,031	12,346,148	26	(12)
60-64 y	329	4,251	6,114,762	69	(33)	329	3,294	14,447,804	24	(10)

SD; standard deviation

[a] These cells are cross-clasified by sex, age (five year categories), and 11 occupations.

[b] Suicide rate was calculated on the basis of the means of the proportion of suicide for each cell type across all prefectures.
