# Supplementary material for: Social and Geographical Inequalities in Suicide in Japan from 1975 through 2005: A Census-Based Longitudinal Analysis
Source: PLoS One. 2013 May 6;8(5):e63443. doi: 10.1371/journal.pone.0063443 (PMC3646025; doi:10.1371/journal.pone.0063443)
Supplement: Table S4 — Time trends in age-adjusted suicide rates per 100,000 in each occupation across eight regions, Japan, 1975–2005. (PDF) [file pone.0063443.s004.pdf]

Table S4. Time trends in age-adjusted suicide rates per 100,000 in each occupation across eight regions, Japan, 1975–2005[a]

	Men				Women			
	Overall	1975–1985	1990–1995	2000–2005	Overall	1975–1985	1990–1995	2000–2005
Hokkaido region (Hokkaido)								
Specialist and technical workers	25	16	16	43	8	16	4	6
Administrative and managerial workers	23	17	18	67	19	18	32	4
Clerical workers	24	24	19	28	5	8	1	5
Sales workers	30	40	22	28	6	9	3	6
Service workers	56	43	38	84	9	9	5	11
Security workers	25	15	25	35	53	159	0	48
Agriculture, forestry and fishery workers	39	37	39	49	14	16	15	4
Transport and communication workers	28	24	22	39	37	27	63	24
Production process and related workers	20	22	15	22	4	5	4	2
Workers not classifiable by occupation	365	2,114	625	210	163	683	261	90
Non-employed [b]	184	191	134	219	18	19	15	21
Tohoku region (Aomori, Iwate, Miyagi, Akita, Yamagata, and Fukushima)								
Specialist and technical workers	31	18	16	58	9	9	7	9
Administrative and managerial workers	25	16	23	60	13	12	20	0
Clerical workers	20	21	18	22	4	6	3	4
Sales workers	29	33	21	30	9	10	8	7
Service workers	63	38	48	98	12	16	9	9
Security workers	26	10	13	49	25	148	0	0
Agriculture, forestry and fishery workers	45	40	43	83	15	15	15	9
Transport and communication workers	29	21	23	47	9	12	7	8
Production process and related workers	24	23	21	29	5	6	5	3
Workers not classifiable by occupation	691	1,907	952	491	212	360	414	124
Non-employed [b]	196	166	176	243	23	22	22	26
Kanto region (Ibaraki, Tochigi, Gunma, Saitama, Chiba, Tokyo, and Kanagawa)								
Specialist and technical workers	19	16	13	27	8	9	8	7
Administrative and managerial workers	18	14	13	40	27	19	31	33
Clerical workers	16	18	15	15	4	5	5	3
Sales workers	15	21	11	15	6	8	5	5
Service workers	37	29	32	48	8	9	8	8
Security workers	19	16	15	26	35	39	30	35
Agriculture, forestry and fishery workers	42	40	42	57	17	19	12	8
Transport and communication workers	24	25	20	29	36	50	20	40
Production process and related workers	15	17	12	13	5	7	4	3
Workers not classifiable by occupation	335	1,022	387	245	123	224	155	90
Non-employed [b]	189	190	171	200	18	18	17	20
Chubu region (Niigata, Toyama, Ishikawa, Fukui, Yamanashi, Nagano, Gifu, Shizuoka, and Aichi)								
Specialist and technical workers	26	19	15	41	8	10	6	7
Administrative and managerial workers	21	18	13	43	22	17	23	32
Clerical workers	17	17	15	19	4	6	4	3
Sales workers	21	25	14	22	7	8	7	5
Service workers	49	28	41	78	8	10	7	8
Security workers	22	14	17	36	50	167	72	8
Agriculture, forestry and fishery workers	46	42	51	62	15	16	15	8
Transport and communication workers	28	24	23	38	24	41	20	20
Production process and related workers	19	22	15	18	4	5	3	3
Workers not classifiable by occupation	606	2,811	1,523	339	173	293	583	58
Non-employed [b]	191	185	162	218	22	22	19	23
Kinki region (Mie, Shiga, Kyoto, Osaka, Hyogo, Nara, and Wakayama)								
Specialist and technical workers	22	18	14	33	7	9	5	6
Administrative and managerial workers	14	10	13	29	27	23	40	17
Clerical workers	20	23	18	20	5	8	4	4
Sales workers	17	23	11	16	7	10	4	4
Service workers	38	29	33	54	8	10	7	6
Security workers	20	20	13	25	55	152	79	12
Agriculture, forestry and fishery workers	36	37	37	35	17	17	14	21
Transport and communication workers	25	24	17	32	26	25	40	20
Production process and related workers	15	19	11	13	5	7	4	3
Workers not classifiable by occupation	529	1,417	589	377	183	264	256	121
Non-employed [b]	171	183	146	177	18	20	15	20
Chugoku region (Tottori, Shimane, Okayama, Hiroshima, and Yamaguchi)								
Specialist and technical workers	25	16	18	41	7	8	8	6
Administrative and managerial workers	18	18	10	25	17	16	14	19
Clerical workers	19	20	17	18	4	7	3	3
Sales workers	22	31	16	19	7	10	5	4
Service workers	45	37	41	56	9	11	7	8
Security workers	20	21	17	24	38	63	0	39
Agriculture, forestry and fishery workers	56	50	81	55	15	15	20	18
Transport and communication workers	23	23	19	29	22	16	17	20
Production process and related workers	18	20	15	17	4	5	3	2
Workers not classifiable by occupation	817	3,761	1,108	532	249	434	418	167
Non-employed [b]	184	190	191	179	22	22	21	23
Shikoku region (Tokushima, Kagawa, Ehime, and Kochi)								
Specialist and technical workers	27	23	16	40	8	10	7	7
Administrative and managerial workers	19	13	17	41	36	56	4	26
Clerical workers	19	20	17	17	4	6	4	3
Sales workers	25	34	16	24	8	9	4	9
Service workers	42	29	26	69	12	13	11	11
Security workers	25	18	26	31	0	0	0	0
Agriculture, forestry and fishery workers	51	49	53	55	16	18	8	8
Transport and communication workers	22	24	10	28	22	18	22	17
Production process and related workers	20	23	17	18	5	6	5	3
Workers not classifiable by occupation	394	701	563	324	167	558	161	77
Non-employed [b]	160	159	146	174	22	23	22	21
Kyushu region (Fukuoka, Saga, Nagasaki, Kumamoto, Oita, Miyazaki, Kagoshima, and Okinawa)								
Specialist and technical workers	28	19	17	45	5	7	5	5
Administrative and managerial workers	17	14	9	36	18	9	25	18
Clerical workers	22	21	23	23	4	5	4	3
Sales workers	29	36	21	29	8	10	7	6
Service workers	55	33	47	83	8	9	8	8
Security workers	23	19	19	31	45	46	33	55
Agriculture, forestry and fishery workers	53	50	54	62	17	18	14	14
Transport and communication workers	29	22	25	41	19	13	25	21
Production process and related workers	21	22	19	22	5	6	5	3
Workers not classifiable by occupation	591	1,492	741	441	168	299	270	116
Non-employed [b]	170	162	156	191	20	19	20	23

[a] Age-adjusted suicide rates were calculated by the direct method, using the model population of 1985 in Japan as a standard. The model population of 1985 is based on the Japanese population under census of 1985 and it is created on the basis of 1,000 persons as 1 unit, after adjusting radical increase or decrease such as baby boom.

[b] Non-employed includes the unemployed as well as the non-labor force.
