# Supplementary material for: Social and Geographical Inequalities in Suicide in Japan from 1975 through 2005: A Census-Based Longitudinal Analysis
Source: PLoS One. 2013 May 6;8(5):e63443. doi: 10.1371/journal.pone.0063443 (PMC3646025; doi:10.1371/journal.pone.0063443)
Supplement: Table S5 — Adjusted prefecture-level residuals for suicide mortality among men, Japan, 1975–2005. (PDF) [file pone.0063443.s005.pdf]

Table S5. Adjusted prefecture-level residuals for suicide mortality among men, Japan, 1975–2005

		Overall			1975			1980			1985			1990			1995			2000			2005		
	**Prefectures**	**OR**	**95% CI**	**Rank**	**OR**	**95% CI**	**Rank**	**OR**	**95% CI**	**Rank**	**OR**	**95% CI**	**Rank**	**OR**	**95% CI**	**Rank**	**OR**	**95% CI**	**Rank**	**OR**	**95% CI**	**Rank**	**OR**	**95% CI**	**Rank**
1	Hokkaido	1.03	0.94, 1.12	30	1.07	0.97, 1.18	39	1.06	0.96, 1.16	34	1.08	0.99, 1.18	36	0.95	0.86, 1.05	19	0.94	0.84, 1.05	19	1.10	1.01, 1.20	36	1.05	0.96, 1.16	28
2	Aomori	1.16	1.05, 1.28	41	0.98	0.86, 1.12	17	1.11	0.97, 1.28	43	1.07	0.93, 1.21	34	1.03	0.88, 1.19	29	1.22	1.04, 1.43	40	1.13	0.99, 1.29	37	1.55	1.36, 1.77	46
3	Iwate	1.24	1.12, 1.37	45	1.09	0.95, 1.25	42	1.18	1.02, 1.36	45	1.13	0.99, 1.29	41	1.07	0.91, 1.25	34	1.42	1.22, 1.66	45	1.31	1.14, 1.50	45	1.36	1.18, 1.57	43
4	Miyagi	0.95	0.86, 1.05	17	0.92	0.81, 1.05	7	0.92	0.80, 1.06	8	0.95	0.84, 1.08	17	0.94	0.81, 1.08	14	0.83	0.71, 0.97	10	0.99	0.88, 1.12	22	1.20	1.06, 1.35	38
5	Akita	1.36	1.23, 1.51	47	1.01	0.88, 1.16	26	1.08	0.93, 1.26	36	1.33	1.16, 1.52	47	1.23	1.05, 1.43	45	1.64	1.40, 1.92	47	1.55	1.35, 1.77	47	1.64	1.42, 1.89	47
6	Yamagata	1.11	1.00, 1.23	39	0.99	0.86, 1.14	19	1.03	0.88, 1.21	31	0.90	0.77, 1.05	10	1.03	0.87, 1.21	30	1.26	1.06, 1.50	44	1.24	1.07, 1.44	43	1.39	1.19, 1.62	45
7	Fukushima	0.98	0.89, 1.09	21	0.89	0.78, 1.02	2	0.87	0.75, 1.00	4	0.97	0.85, 1.10	19	0.86	0.74, 0.99	2	1.07	0.92, 1.25	33	1.09	0.96, 1.23	33	1.38	1.21, 1.56	44
8	Ibaraki	0.94	0.85, 1.03	15	1.01	0.90, 1.14	27	0.98	0.86, 1.11	22	0.93	0.83, 1.05	15	0.86	0.75, 0.99	3	0.90	0.78, 1.04	15	1.00	0.90, 1.12	25	0.95	0.84, 1.08	21
9	Tochigi	1.00	0.91, 1.10	23	0.96	0.84, 1.10	16	1.01	0.88, 1.17	28	1.06	0.93, 1.20	33	1.04	0.90, 1.21	33	1.16	0.99, 1.34	36	0.93	0.81, 1.05	14	0.93	0.80, 1.07	18
10	Gunma	1.00	0.91, 1.11	24	0.99	0.87, 1.13	21	1.09	0.95, 1.25	39	1.07	0.94, 1.21	35	0.93	0.80, 1.08	12	1.06	0.91, 1.23	30	0.88	0.77, 1.00	10	1.04	0.91, 1.20	25
11	Saitama	0.84	0.77, 0.92	4	0.91	0.82, 1.01	4	0.91	0.82, 1.01	6	0.87	0.79, 0.96	8	0.89	0.80, 0.99	6	0.82	0.74, 0.92	8	0.78	0.71, 0.85	5	0.71	0.64, 0.78	3
12	Chiba	0.85	0.77, 0.93	5	0.92	0.83, 1.02	8	0.84	0.75, 0.94	2	0.83	0.75, 0.91	2	0.87	0.78, 0.96	4	0.80	0.71, 0.89	6	0.84	0.77, 0.91	8	0.81	0.73, 0.90	7
13	Tokyo	0.86	0.79, 0.94	6	0.95	0.88, 1.02	13	0.92	0.85, 1.00	9	0.82	0.76, 0.88	1	0.90	0.83, 0.98	8	0.78	0.72, 0.86	2	0.78	0.72, 0.83	4	0.73	0.67, 0.80	4
14	Kanagawa	0.83	0.76, 0.90	3	0.89	0.81, 0.98	1	0.75	0.68, 0.83	1	0.87	0.79, 0.95	7	0.89	0.81, 0.98	7	0.79	0.71, 0.87	4	0.83	0.76, 0.89	7	0.69	0.63, 0.76	2
15	Niigata	1.22	1.11, 1.34	43	1.10	0.97, 1.25	43	1.10	0.97, 1.25	42	1.21	1.08, 1.35	44	1.32	1.16, 1.50	47	1.24	1.08, 1.42	42	1.35	1.21, 1.51	46	1.24	1.10, 1.40	41
16	Toyama	1.17	1.05, 1.29	42	0.96	0.83, 1.12	15	1.09	0.93, 1.29	40	1.22	1.05, 1.42	45	1.24	1.04, 1.47	46	1.21	1.00, 1.46	38	1.10	0.94, 1.29	35	1.23	1.04, 1.45	40
17	Ishikawa	1.05	0.94, 1.17	34	1.04	0.89, 1.20	35	1.11	0.95, 1.31	44	1.03	0.88, 1.20	27	1.07	0.90, 1.27	36	0.92	0.75, 1.13	16	0.98	0.84, 1.15	21	1.15	0.98, 1.36	36
18	Fukui	1.01	0.90, 1.13	27	0.93	0.79, 1.10	10	0.98	0.82, 1.17	23	0.98	0.82, 1.17	20	0.97	0.80, 1.18	24	1.12	0.90, 1.38	35	1.13	0.95, 1.35	38	1.08	0.88, 1.31	32
19	Yamanashi	1.07	0.96, 1.20	37	1.00	0.86, 1.17	25	0.94	0.78, 1.13	11	0.99	0.84, 1.18	22	1.07	0.89, 1.28	35	1.10	0.90, 1.36	34	1.21	1.02, 1.42	41	1.21	1.01, 1.44	39
20	Nagano	1.01	0.92, 1.12	28	1.02	0.90, 1.16	32	0.97	0.85, 1.12	21	1.05	0.92, 1.19	31	0.99	0.85, 1.14	26	1.00	0.85, 1.17	27	0.99	0.87, 1.13	23	1.15	1.01, 1.31	35
21	Gifu	1.01	0.91, 1.12	26	1.12	0.98, 1.29	44	0.95	0.82, 1.11	17	1.03	0.90, 1.18	29	1.03	0.89, 1.20	32	0.84	0.70, 0.99	11	1.03	0.90, 1.17	29	1.06	0.92, 1.22	29
22	Shizuoka	0.98	0.89, 1.07	19	1.07	0.95, 1.19	37	1.04	0.93, 1.17	32	1.01	0.90, 1.12	24	0.96	0.85, 1.09	23	0.94	0.83, 1.08	20	0.93	0.84, 1.03	16	0.93	0.82, 1.04	19
23	Aichi	0.89	0.82, 0.98	9	1.01	0.92, 1.11	28	0.86	0.78, 0.96	3	0.86	0.78, 0.94	5	0.89	0.80, 0.98	5	0.93	0.83, 1.03	17	0.90	0.83, 0.98	11	0.87	0.78, 0.96	13
24	Mie	0.88	0.79, 0.97	8	0.90	0.78, 1.04	3	0.95	0.82, 1.10	14	0.92	0.80, 1.06	13	0.95	0.81, 1.10	18	0.88	0.74, 1.04	13	0.94	0.82, 1.07	17	0.82	0.70, 0.96	10
25	Shiga	0.91	0.82, 1.01	10	0.99	0.85, 1.15	22	0.92	0.78, 1.10	10	1.00	0.85, 1.17	23	0.96	0.80, 1.14	20	0.98	0.81, 1.19	23	0.81	0.69, 0.95	6	0.89	0.75, 1.06	14
26	Kyoto	0.92	0.84, 1.02	11	1.02	0.91, 1.16	34	1.01	0.89, 1.15	26	0.89	0.78, 1.00	9	0.94	0.82, 1.08	17	0.82	0.71, 0.95	9	0.92	0.82, 1.04	13	0.84	0.74, 0.97	12
27	Osaka	0.93	0.85, 1.01	13	1.16	1.07, 1.26	47	0.96	0.88, 1.04	18	0.85	0.78, 0.92	3	0.94	0.86, 1.03	16	0.79	0.71, 0.87	3	0.95	0.88, 1.03	18	0.73	0.67, 0.81	5
28	Hyogo	0.93	0.84, 1.01	12	1.04	0.94, 1.14	36	1.03	0.93, 1.14	30	0.92	0.84, 1.01	14	0.90	0.81, 1.00	9	0.81	0.72, 0.91	7	0.88	0.80, 0.96	9	0.83	0.74, 0.92	11
29	Nara	0.76	0.68, 0.85	1	0.92	0.78, 1.07	5	0.91	0.77, 1.08	7	0.85	0.72, 0.99	4	0.91	0.77, 1.08	11	0.63	0.51, 0.78	1	0.71	0.61, 0.84	1	0.67	0.55, 0.80	1
30	Wakayama	1.00	0.90, 1.12	25	0.93	0.80, 1.08	9	0.97	0.83, 1.14	20	1.22	1.06, 1.41	46	0.96	0.81, 1.14	22	0.96	0.80, 1.17	21	1.00	0.86, 1.17	26	1.01	0.85, 1.20	23
31	Tottori	1.06	0.94, 1.19	35	0.95	0.81, 1.12	14	1.08	0.90, 1.30	35	0.93	0.77, 1.13	16	1.10	0.90, 1.34	38	1.22	0.98, 1.53	41	0.98	0.81, 1.19	20	1.18	0.96, 1.45	37
32	Shimane	1.27	1.14, 1.42	46	1.09	0.93, 1.27	41	1.30	1.08, 1.55	47	1.16	0.98, 1.38	43	1.17	0.97, 1.40	42	1.54	1.26, 1.88	46	1.22	1.02, 1.45	42	1.07	0.87, 1.30	31
33	Okayama	0.87	0.79, 0.97	7	1.00	0.88, 1.14	24	0.99	0.86, 1.14	25	0.90	0.79, 1.03	11	0.98	0.84, 1.13	25	0.88	0.74, 1.04	12	0.76	0.66, 0.88	3	0.78	0.67, 0.92	6
34	Hiroshima	0.97	0.88, 1.07	18	0.99	0.88, 1.11	18	0.98	0.87, 1.12	24	1.03	0.92, 1.15	28	1.11	0.98, 1.25	39	1.00	0.87, 1.14	26	0.93	0.83, 1.04	15	0.82	0.72, 0.94	9
35	Yamaguchi	1.07	0.97, 1.18	36	1.14	0.99, 1.32	46	1.09	0.95, 1.26	41	1.10	0.96, 1.25	38	1.12	0.97, 1.31	40	0.99	0.83, 1.17	24	1.05	0.91, 1.20	31	0.92	0.78, 1.08	17
36	Tokushima	0.81	0.72, 0.91	2	0.95	0.81, 1.10	11	0.96	0.81, 1.14	19	0.86	0.73, 1.02	6	0.83	0.69, 1.01	1	0.80	0.64, 0.99	5	0.74	0.62, 0.90	2	0.82	0.67, 1.00	8
37	Kagawa	0.95	0.85, 1.06	16	1.07	0.92, 1.24	38	0.95	0.80, 1.12	12	0.96	0.82, 1.13	18	0.96	0.81, 1.14	21	0.88	0.72, 1.09	14	0.96	0.81, 1.13	19	0.94	0.78, 1.12	20
38	Ehime	1.00	0.90, 1.10	22	1.02	0.89, 1.16	29	1.01	0.87, 1.17	27	1.02	0.89, 1.17	25	1.01	0.87, 1.18	28	1.04	0.88, 1.24	29	1.02	0.88, 1.17	28	0.92	0.78, 1.07	16
39	Kochi	1.05	0.94, 1.17	33	1.07	0.92, 1.25	40	1.05	0.89, 1.24	33	1.10	0.95, 1.28	39	0.99	0.83, 1.18	27	0.93	0.76, 1.14	18	1.01	0.85, 1.20	27	1.04	0.87, 1.25	26
40	Fukuoka	1.03	0.94, 1.13	31	1.02	0.92, 1.12	30	0.95	0.86, 1.05	15	1.02	0.93, 1.12	26	1.08	0.98, 1.20	37	1.06	0.95, 1.18	31	1.04	0.95, 1.13	30	1.04	0.94, 1.15	27
41	Saga	1.09	0.97, 1.21	38	1.00	0.85, 1.16	23	1.09	0.92, 1.29	37	1.11	0.94, 1.31	40	1.03	0.86, 1.24	31	1.07	0.87, 1.32	32	1.18	1.00, 1.39	39	1.01	0.84, 1.22	24
42	Nagasaki	1.04	0.94, 1.15	32	1.02	0.89, 1.16	31	0.95	0.82, 1.10	13	1.05	0.93, 1.20	32	0.93	0.80, 1.09	13	1.01	0.86, 1.20	28	1.10	0.96, 1.25	34	1.25	1.08, 1.44	42
43	Kumamoto	0.94	0.85, 1.03	14	0.92	0.80, 1.05	6	0.95	0.83, 1.10	16	0.98	0.86, 1.11	21	0.90	0.78, 1.05	10	0.97	0.83, 1.14	22	0.91	0.80, 1.04	12	1.06	0.92, 1.22	30
44	Oita	0.98	0.88, 1.09	20	0.99	0.86, 1.14	20	1.03	0.88, 1.20	29	1.05	0.90, 1.21	30	0.94	0.80, 1.11	15	1.00	0.83, 1.20	25	0.99	0.86, 1.16	24	0.90	0.76, 1.07	15
45	Miyazaki	1.23	1.11, 1.36	44	1.14	0.98, 1.32	45	1.29	1.10, 1.51	46	1.09	0.94, 1.25	37	1.21	1.04, 1.42	43	1.22	1.02, 1.45	39	1.27	1.10, 1.46	44	1.13	0.96, 1.32	34
46	Kagoshima	1.13	1.03, 1.25	40	1.02	0.90, 1.16	33	1.09	0.95, 1.25	38	1.14	1.00, 1.29	42	1.14	0.99, 1.31	41	1.25	1.07, 1.45	43	1.18	1.04, 1.34	40	1.10	0.95, 1.26	33
47	Okinawa	1.02	0.92, 1.13	29	0.95	0.82, 1.10	12	0.89	0.75, 1.05	5	0.90	0.78, 1.05	12	1.22	1.05, 1.42	44	1.19	1.01, 1.39	37	1.06	0.93, 1.21	32	0.96	0.83, 1.12	22

CI; credible interval, OR; odds ratio

Prefectures with a lower estimate of odds for suicide mortality are ranked higher. The reference is the grand mean of all prefectures.
