# Supplementary material for: Social and Geographical Inequalities in Suicide in Japan from 1975 through 2005: A Census-Based Longitudinal Analysis
Source: PLoS One. 2013 May 6;8(5):e63443. doi: 10.1371/journal.pone.0063443 (PMC3646025; doi:10.1371/journal.pone.0063443)
Supplement: Table S6 — Adjusted prefecture-level residuals for suicide mortality among women, Japan, 1975–2005. (PDF) [file pone.0063443.s006.pdf]

Table S6. Adjusted prefecture-level residuals for suicide mortality among women, Japan, 1975–2005

	Overall			1975			1980			1985			1990			1995			2000			2005		
Prefectures	OR	95% CI	Rank	OR	95% CI	Rank	OR	95% CI	Rank	OR	95% CI	Rank	OR	95% CI	Rank	OR	95% CI	Rank	OR	95% CI	Rank	OR	95% CI	Rank
1 Hokkaido	0.92	0.85, 0.99	9	1.04	0.93, 1.16	34	0.92	0.82, 1.04	7	0.98	0.86, 1.11	20	0.84	0.73, 0.96	2	0.83	0.71, 0.96	5	0.95	0.84, 1.07	17	1.02	0.90, 1.15	32
2 Aomori	1.02	0.93, 1.13	30	0.92	0.78, 1.08	6	1.06	0.90, 1.24	38	1.01	0.83, 1.23	24	1.09	0.91, 1.30	40	0.97	0.78, 1.21	20	1.11	0.92, 1.33	40	1.01	0.83, 1.21	29
3 Iwate	1.18	1.07, 1.31	46	1.12	0.95, 1.33	46	0.98	0.83, 1.16	16	1.18	0.97, 1.44	44	1.02	0.85, 1.22	29	1.24	0.99, 1.54	45	1.14	0.94, 1.38	43	1.21	0.99, 1.49	47
4 Miyagi	0.92	0.83, 1.01	10	1.00	0.86, 1.17	21	0.91	0.77, 1.07	5	1.01	0.85, 1.21	23	0.92	0.77, 1.10	9	1.01	0.83, 1.24	24	0.92	0.77, 1.10	10	0.91	0.76, 1.08	6
5 Akita	1.22	1.10, 1.35	47	1.06	0.90, 1.25	40	1.04	0.88, 1.23	34	1.26	1.04, 1.54	45	1.05	0.87, 1.26	36	1.29	1.03, 1.62	47	1.22	0.99, 1.50	46	1.15	0.94, 1.40	45
6 Yamagata	1.05	0.94, 1.17	34	1.01	0.85, 1.19	23	1.09	0.92, 1.30	44	1.06	0.86, 1.30	30	0.96	0.79, 1.17	14	1.14	0.90, 1.44	38	0.93	0.76, 1.15	12	1.08	0.88, 1.32	37
7 Fukushima	1.02	0.93, 1.12	29	0.88	0.74, 1.04	2	0.99	0.84, 1.16	19	1.11	0.93, 1.33	36	1.09	0.92, 1.29	41	1.09	0.89, 1.33	33	0.98	0.82, 1.18	23	1.11	0.93, 1.32	41
8 Ibaraki	0.98	0.90, 1.07	19	0.93	0.80, 1.08	10	1.01	0.87, 1.17	26	1.15	0.98, 1.36	41	1.04	0.89, 1.22	34	0.94	0.78, 1.13	18	0.89	0.76, 1.05	5	0.99	0.85, 1.16	26
9 Tochigi	1.01	0.92, 1.12	28	0.91	0.77, 1.07	5	1.07	0.91, 1.27	42	1.14	0.94, 1.37	40	1.12	0.94, 1.34	43	0.90	0.72, 1.13	13	1.09	0.92, 1.30	38	0.90	0.75, 1.09	5
10 Gunma	1.14	1.03, 1.25	42	0.99	0.85, 1.15	17	1.13	0.96, 1.33	46	1.28	1.07, 1.54	47	1.10	0.92, 1.31	42	1.20	0.98, 1.46	44	1.08	0.91, 1.28	37	0.97	0.81, 1.17	17
11 Saitama	0.91	0.84, 0.98	7	0.98	0.87, 1.11	16	1.02	0.90, 1.15	27	0.81	0.71, 0.94	2	0.98	0.87, 1.11	18	0.91	0.79, 1.05	15	0.88	0.79, 1.00	4	0.89	0.78, 1.00	4
12 Chiba	0.82	0.75, 0.89	2	0.88	0.77, 1.01	3	0.83	0.72, 0.96	2	0.83	0.72, 0.96	6	0.84	0.72, 0.96	1	0.77	0.66, 0.90	2	0.89	0.79, 1.01	7	0.91	0.80, 1.03	7
13 Tokyo	0.97	0.91, 1.04	17	0.99	0.90, 1.09	19	0.90	0.82, 1.00	3	0.93	0.84, 1.04	15	0.96	0.86, 1.06	12	0.90	0.80, 1.01	12	1.03	0.93, 1.13	29	1.09	0.99, 1.20	39
14 Kanagawa	0.79	0.74, 0.86	1	0.86	0.76, 0.97	1	0.77	0.67, 0.88	1	0.76	0.67, 0.87	1	0.90	0.80, 1.02	5	0.80	0.70, 0.91	3	0.87	0.78, 0.98	3	0.80	0.71, 0.91	1
15 Niigata	1.15	1.05, 1.26	43	1.04	0.90, 1.20	33	1.07	0.92, 1.24	40	1.08	0.91, 1.28	33	1.18	1.00, 1.40	46	1.16	0.96, 1.41	41	1.26	1.06, 1.49	47	1.04	0.88, 1.23	34
16 Toyama	1.18	1.05, 1.32	44	0.99	0.84, 1.17	18	1.02	0.86, 1.22	29	1.08	0.87, 1.35	35	1.13	0.93, 1.39	44	1.16	0.91, 1.47	40	1.16	0.94, 1.44	45	1.20	0.97, 1.48	46
17 Ishikawa	1.00	0.89, 1.13	25	1.03	0.87, 1.22	32	1.02	0.85, 1.21	28	1.07	0.86, 1.33	32	0.98	0.80, 1.19	17	1.03	0.80, 1.31	25	0.92	0.74, 1.13	9	1.02	0.83, 1.24	31
18 Fukui	0.98	0.86, 1.12	21	1.00	0.84, 1.20	22	0.95	0.79, 1.15	8	0.82	0.63, 1.07	4	1.01	0.82, 1.25	26	1.12	0.86, 1.45	37	1.00	0.80, 1.24	26	1.08	0.87, 1.34	38
19 Yamanashi	1.04	0.92, 1.18	33	1.02	0.85, 1.21	27	1.02	0.85, 1.23	30	1.17	0.92, 1.48	42	1.05	0.85, 1.29	35	1.08	0.83, 1.39	31	0.91	0.73, 1.13	8	0.98	0.80, 1.21	20
20 Nagano	1.08	0.98, 1.19	37	1.05	0.90, 1.22	38	0.92	0.78, 1.09	6	1.12	0.94, 1.34	38	1.14	0.96, 1.36	45	1.17	0.96, 1.44	42	1.11	0.94, 1.32	41	0.99	0.83, 1.18	24
21 Gifu	1.06	0.96, 1.16	35	1.09	0.93, 1.27	43	1.05	0.89, 1.23	36	1.12	0.93, 1.35	37	1.00	0.84, 1.19	23	0.96	0.77, 1.18	19	0.94	0.78, 1.12	13	1.12	0.94, 1.33	42
22 Shizuoka	0.88	0.81, 0.96	4	0.97	0.85, 1.11	14	0.98	0.85, 1.13	14	0.82	0.69, 0.97	3	1.01	0.87, 1.17	25	0.76	0.62, 0.93	1	0.86	0.74, 1.01	2	0.98	0.84, 1.14	19
23 Aichi	0.94	0.87, 1.02	13	1.10	0.98, 1.23	45	1.00	0.89, 1.12	22	0.89	0.78, 1.02	11	0.92	0.81, 1.05	10	0.98	0.85, 1.13	22	0.94	0.83, 1.06	14	0.88	0.77, 1.00	3
24 Mie	0.89	0.80, 0.99	5	0.92	0.78, 1.09	7	1.00	0.85, 1.18	21	0.90	0.73, 1.10	12	0.92	0.77, 1.10	8	0.85	0.68, 1.07	6	0.97	0.81, 1.16	20	0.99	0.83, 1.19	25
25 Shiga	0.91	0.81, 1.02	6	1.01	0.85, 1.20	25	1.03	0.87, 1.23	32	0.82	0.65, 1.05	5	0.98	0.81, 1.20	19	0.83	0.64, 1.07	4	0.92	0.76, 1.12	11	0.97	0.80, 1.18	16
26 Kyoto	0.98	0.89, 1.07	18	1.02	0.89, 1.18	29	1.01	0.87, 1.17	25	0.98	0.83, 1.16	19	0.96	0.82, 1.13	13	0.94	0.78, 1.13	16	0.96	0.82, 1.13	19	0.99	0.84, 1.16	23
27 Osaka	0.93	0.87, 1.00	11	1.20	1.08, 1.32	47	0.95	0.86, 1.06	9	0.84	0.75, 0.95	7	0.86	0.76, 0.96	4	0.86	0.75, 0.97	8	0.94	0.84, 1.04	15	0.92	0.82, 1.03	9
28 Hyogo	0.95	0.88, 1.02	15	1.05	0.94, 1.18	39	1.10	0.98, 1.23	45	0.86	0.75, 0.99	9	0.90	0.79, 1.03	6	0.87	0.75, 1.01	9	0.89	0.79, 1.01	6	1.01	0.89, 1.14	28
29 Nara	0.84	0.75, 0.94	3	1.02	0.86, 1.20	28	1.00	0.85, 1.19	23	0.87	0.70, 1.08	10	0.84	0.69, 1.03	3	0.88	0.70, 1.10	10	0.80	0.65, 0.99	1	0.88	0.72, 1.07	2
30 Wakayama	1.07	0.96, 1.19	36	1.07	0.91, 1.27	41	1.06	0.89, 1.26	39	1.07	0.86, 1.32	31	0.91	0.75, 1.11	7	1.10	0.87, 1.38	35	0.97	0.80, 1.19	21	1.09	0.90, 1.33	40
31 Tottori	1.00	0.87, 1.14	24	0.96	0.80, 1.16	13	0.98	0.80, 1.18	13	1.03	0.80, 1.33	27	1.01	0.82, 1.26	27	1.04	0.79, 1.37	27	1.05	0.84, 1.32	33	0.98	0.79, 1.23	22
32 Shimane	1.10	0.97, 1.24	40	1.03	0.86, 1.23	30	1.05	0.87, 1.27	37	1.13	0.90, 1.44	39	1.05	0.85, 1.30	37	1.08	0.82, 1.41	30	1.08	0.86, 1.34	35	0.95	0.76, 1.18	11
33 Okayama	0.94	0.85, 1.05	14	0.90	0.77, 1.06	4	1.03	0.88, 1.21	31	0.85	0.69, 1.04	8	1.06	0.89, 1.26	38	0.94	0.76, 1.16	17	1.03	0.86, 1.22	30	0.97	0.81, 1.16	13
34 Hiroshima	1.01	0.92, 1.10	26	1.10	0.95, 1.27	44	1.03	0.89, 1.20	33	0.96	0.81, 1.14	17	1.04	0.89, 1.22	32	1.03	0.86, 1.23	26	0.98	0.84, 1.14	22	0.91	0.77, 1.08	8
35 Yamaguchi	1.09	0.99, 1.21	39	1.04	0.89, 1.22	36	1.08	0.92, 1.28	43	1.08	0.89, 1.31	34	0.94	0.78, 1.14	11	1.15	0.93, 1.42	39	1.04	0.86, 1.25	32	1.13	0.94, 1.37	44
36 Tokushima	0.99	0.88, 1.12	22	1.01	0.85, 1.20	24	1.00	0.83, 1.19	20	1.03	0.82, 1.29	26	1.04	0.85, 1.27	31	0.98	0.75, 1.27	21	0.94	0.76, 1.17	16	0.98	0.80, 1.21	21
37 Kagawa	1.03	0.92, 1.15	31	1.04	0.88, 1.24	37	0.98	0.82, 1.18	17	1.02	0.82, 1.28	25	1.07	0.88, 1.30	39	1.09	0.86, 1.39	34	0.96	0.78, 1.18	18	0.98	0.80, 1.20	18
38 Ehime	1.03	0.93, 1.15	32	1.00	0.85, 1.17	20	1.00	0.85, 1.18	24	1.17	0.97, 1.42	43	1.02	0.85, 1.22	28	1.00	0.80, 1.25	23	1.02	0.85, 1.23	27	1.01	0.84, 1.21	30
39 Kochi	1.08	0.96, 1.22	38	1.01	0.85, 1.21	26	1.05	0.87, 1.25	35	1.05	0.83, 1.32	29	0.99	0.80, 1.21	20	1.08	0.84, 1.40	32	1.03	0.83, 1.27	31	1.12	0.91, 1.39	43
40 Fukuoka	0.94	0.87, 1.02	12	0.93	0.82, 1.05	8	0.97	0.86, 1.10	11	0.97	0.84, 1.11	18	0.99	0.87, 1.13	21	0.91	0.78, 1.06	14	0.98	0.87, 1.12	24	0.96	0.84, 1.10	12
41 Saga	0.99	0.88, 1.12	23	0.95	0.79, 1.14	11	0.97	0.81, 1.17	12	0.91	0.71, 1.16	14	1.04	0.85, 1.27	33	1.07	0.83, 1.38	28	1.08	0.87, 1.33	36	1.02	0.83, 1.26	33
42 Nagasaki	0.96	0.86, 1.06	16	0.98	0.84, 1.15	15	0.98	0.84, 1.16	18	0.95	0.78, 1.16	16	0.97	0.82, 1.17	15	0.90	0.71, 1.13	11	1.10	0.92, 1.33	39	0.97	0.81, 1.17	14
43 Kumamoto	1.01	0.92, 1.12	27	1.03	0.88, 1.20	31	0.96	0.82, 1.13	10	0.91	0.75, 1.11	13	0.99	0.83, 1.18	22	1.11	0.91, 1.37	36	1.05	0.88, 1.25	34	1.07	0.90, 1.28	36
44 Oita	0.98	0.88, 1.10	20	0.95	0.80, 1.12	12	0.98	0.83, 1.17	15	1.00	0.81, 1.23	22	0.98	0.81, 1.18	16	1.07	0.85, 1.35	29	1.12	0.93, 1.36	42	0.92	0.76, 1.13	10
45 Miyazaki	1.13	1.01, 1.26	41	1.04	0.88, 1.23	35	1.07	0.90, 1.28	41	1.00	0.81, 1.24	21	1.02	0.84, 1.23	30	1.28	1.01, 1.62	46	1.16	0.95, 1.42	44	1.05	0.86, 1.29	35
46 Kagoshima	1.18	1.07, 1.29	45	1.08	0.92, 1.26	42	1.14	0.97, 1.34	47	1.27	1.07, 1.52	46	1.23	1.03, 1.47	47	1.18	0.96, 1.44	43	1.02	0.85, 1.23	28	0.97	0.81, 1.16	15
47 Okinawa	0.91	0.81, 1.02	8	0.93	0.78, 1.12	9	0.91	0.75, 1.09	4	1.04	0.84, 1.29	28	1.01	0.83, 1.22	24	0.85	0.66, 1.09	7	0.99	0.82, 1.20	25	1.00	0.82, 1.20	27

CI; credible interval, OR; odds ratio

Prefectures with a lower estimate of odds for suicide mortality are ranked higher. The reference is the grand mean of all prefectures.
