# Supplementary material for: Social and Geographical Inequalities in Suicide in Japan from 1975 through 2005: A Census-Based Longitudinal Analysis
Source: PLoS One. 2013 May 6;8(5):e63443. doi: 10.1371/journal.pone.0063443 (PMC3646025; doi:10.1371/journal.pone.0063443)
Supplement: Table S7 — The number of deaths classified as undetermined intent among those aged 25–64 years, Japan, 1975–2005. (PDF) [file pone.0063443.s007.pdf]

**Table S7.** The number of deaths classified as undetermined intent among those aged 25–64 years, Japan, 1975–2005[a]

| | 1975 | | 1980 | | 1985 | | 1990 | | 1995 | | 2000 | | 2005 | |
|---|---|---|---|---|---|---|---|---|---|---|---|---|---|---|
| | **N** | **%** [b] | **N** | **%** | **N** | **%** | **N** | **%** | **N** | **%** | **N** | **%** | **N** | **%** |
| Men | 546 | 6.63 | 850 | 8.65 | 1,030 | 8.34 | 1,117 | 11.53 | 616 | 5.73 | 875 | 5.26 | 882 | 5.17 |
| Women | 200 | 4.19 | 288 | 6.16 | 330 | 6.67 | 437 | 9.45 | 234 | 5.68 | 350 | 6.79 | 374 | 7.20 |

[a] In the Vital Statistics, event of undetermined intent was coded according to the International Classification of Disease (ICD) as follows: ICD-8 codes: E980–E989 in 1975; ICD-9 codes: E980–E989 in 1980 to 1990; and ICD-10 codes: Y10–Y34 in 1995 to 2005.

[b] The percentages of deaths classified as undetermined intent among certified suicides and undetermined deaths combined are shown. In the Vital Statistics, suicide was coded according to the ICD as follows: ICD-8 codes: E950–E959 in 1975; ICD-9 codes: E950–E959 in 1980 to 1990; and ICD-10 codes: X60–X84 in 1995 to 2005.
